# Hierarchical Icephobic Surfaces with Enhanced Photothermal Performance for Sustainable Anti‐Icing

**DOI:** 10.1002/advs.202502945

**Published:** 2025-05-08

**Authors:** Lei Zhang, Yongle Feng, Xixin Cao, Yue Dong, Weihong Liu, Bing Li, Jing Li, Chonglei Hao

**Affiliations:** ^1^ School of Mechanical Engineering and Automation Harbin Institute of Technology Shenzhen 518055 China; ^2^ School of Materials Science and Engineering Harbin Institute of Technology Shenzhen 518055 China; ^3^ Department of Materials Science and Engineering City University of Hong Kong Hong Kong SAR 999077 China

**Keywords:** armored silica shell, durability, hierarchical micro‐nanostructures, icephobic surfaces, photothermal effects

## Abstract

Icing remains a major challenge in industrial and environmental applications, leading to efficiency losses, safety hazards, and substantial economic impacts. Conventional deicing methods are energy‐intensive and environmentally unsustainable, often requiring high energy inputs, extensive operational maintenance, or the use of harmful chemicals. These drawbacks underscore the need for advanced, scalable solutions that are both efficient and environmentally responsible. Here, the armored photothermal icephobic structured surface (APISS) is presented that combines superhydrophobicity and photothermal effects to deliver superior anti‐icing performance. The APISS consists hierarchical micro‐nanostructures with titanium nitride (TiN) nanoparticles encapsulated in a silica shell, ensuring exceptional durability and efficient solar energy conversion. Under 1 sun illumination, APISS achieves a temperature increase of 35 °C, effectively melting ice within 179 s and preventing refreezing. Its superhydrophobic properties facilitate the removal of melted water, maintaining a clean and dry surface. Comprehensive testing reveals that APISS significantly outperforms existing anti‐icing materials in scalability, durability, and sustainability, making it highly suitable for renewable energy, aviation, and infrastructure maintenance. The work highlights APISS as an advanced approach to anti‐icing technology, addressing critical challenges with a scalable and environmentally friendly solution.

## Graphical abstract

The armored photothermal icephobic structured surface (APISS) features a hierarchical micro‐nanostructured architecture optimized for enhanced solar absorption, coated with titanium nitride (TiN) nanoparticles to maximize photothermal conversion efficiency and silica encapsulation to ensure exceptional mechanical resilience. It offers a robust, efficient, and sustainable solution for mitigating icing across diverse applications.

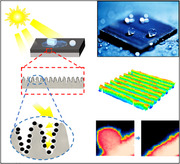

## 1 Introduction

Icing presents a persistent and multifaceted challenge across a wide spectrum of industrial and environmental applications, exerting significant operational and safety constraints on infrastructure, transportation, and energy systems.^[^ ^1^, ^2^, ^3^, ^4^ ^]^ Ice accretion on wind turbine blades can severely reduce energy generation efficiency,^[^ ^5^, ^6^ ^]^ with reported losses of up to 50% in cold climates,^[^ ^7^ ^]^ while frost accumulation on aircraft surfaces and power transmission lines compromises safety and incurs substantial economic costs.^[^ ^8^, ^9^, ^10^ ^]^ Traditional methods for mitigating icing, including mechanical removal, chemical deicing agents, and electrical heating systems, are typically energy‐intensive, environmentally harmful, and financially prohibitive.^[^ ^11^, ^12^, ^13^ ^]^ These limitations underscore the urgent need for more efficient and sustainable anti‐icing solutions.

Advances in materials science have catalyzed the emergence of innovative anti‐icing technologies, broadly categorized into passive and active strategies.^[^ ^14^, ^15^, ^16^, ^17^ ^]^ Among passive approaches, superhydrophobic surfaces (SHSs), inspired by the lotus leaf, reduce ice adhesion and delay freezing by minimizing the contact area between water droplets and the substrate.^[^ ^18^, ^19^, ^20^, ^21^, ^22^ ^]^ However, SHSs often suffer performance degradation under conditions of high humidity or dynamic freezing, as their micro‐nano hierarchical structures are inherently unstable.^[^ ^23^, ^24^, ^25^ ^]^ Similarly, slippery liquid‐infused porous surfaces (SLIPSs) provide icephobicity through the formation of a lubricant film that prevents ice anchoring.^[^ ^26^, ^27^, ^28^, ^29^ ^]^ Yet, these surfaces are prone to lubricant depletion through evaporation or environmental exposure, limiting their long‐term utility in harsh, real‐world scenarios.^[^ ^30^ ^]^ Active anti‐icing technologies, particularly those leveraging photothermal effects, have gained considerable attention for their ability to harness solar energy to generate localized heating, melting ice, and preventing reformation.^[^ ^31^, ^32^, ^33^ ^]^ Photothermal surfaces achieve this by converting absorbed light into thermal energy, which reduces ice adhesion by forming a liquid interfacial layer. While promising, photothermal materials such as plasmonic nanoparticles,^[^ ^34^, ^35^, ^36^ ^]^ carbon‐based systems,^[^ ^37^, ^38^, ^39^ ^]^ and metallic coatings face critical limitations,^[^ ^32^, ^40^, ^41^ ^]^ including high production costs, scalability challenges, and durability concerns under prolonged environmental stress. Furthermore, these materials often fail to address the problem of residual melted water, which can refreeze and undermine long‐term anti‐icing effectiveness.

To overcome these challenges, we have developed an armored photothermal ice‐phobic structured surface (APISS), which synergistically integrates superhydrophobicity and photothermal effects for robust, reliable, and sustainable anti‐icing performance. The APISS features a hierarchical micro‐nanostructured architecture optimized for enhanced solar absorption, coated with titanium nitride (TiN) nanoparticles to maximize photothermal conversion efficiency and silica encapsulation to ensure exceptional mechanical resilience.^[^ ^42^ ^]^ Such a multiscale and multifunctional integration enables near‐complete solar absorption and significant localized heating under ambient light conditions. Meanwhile, its superhydrophobic nature facilitates the rapid removal of melted water, mitigating refreezing risks and maintaining a clean, dry surface. Through comprehensive investigations of the optical, thermal, and mechanical properties of the APISS, we elucidated the mechanisms underpinning its exceptional performance and explore its potential for deployment in demanding, real‐world environments. This work demonstrates how the strategic integration of superhydrophobicity and photothermal effects addresses the limitations of existing anti‐icing strategies, offering a scalable, cost‐effective, and environmentally sustainable solution suitable for a diverse range of applications, including renewable energy systems, aviation, and infrastructure in cold climates.

## 2 Results

### 2.1 Design, Fabrication, and Structure of the APISS Coating

**Figure 1:**
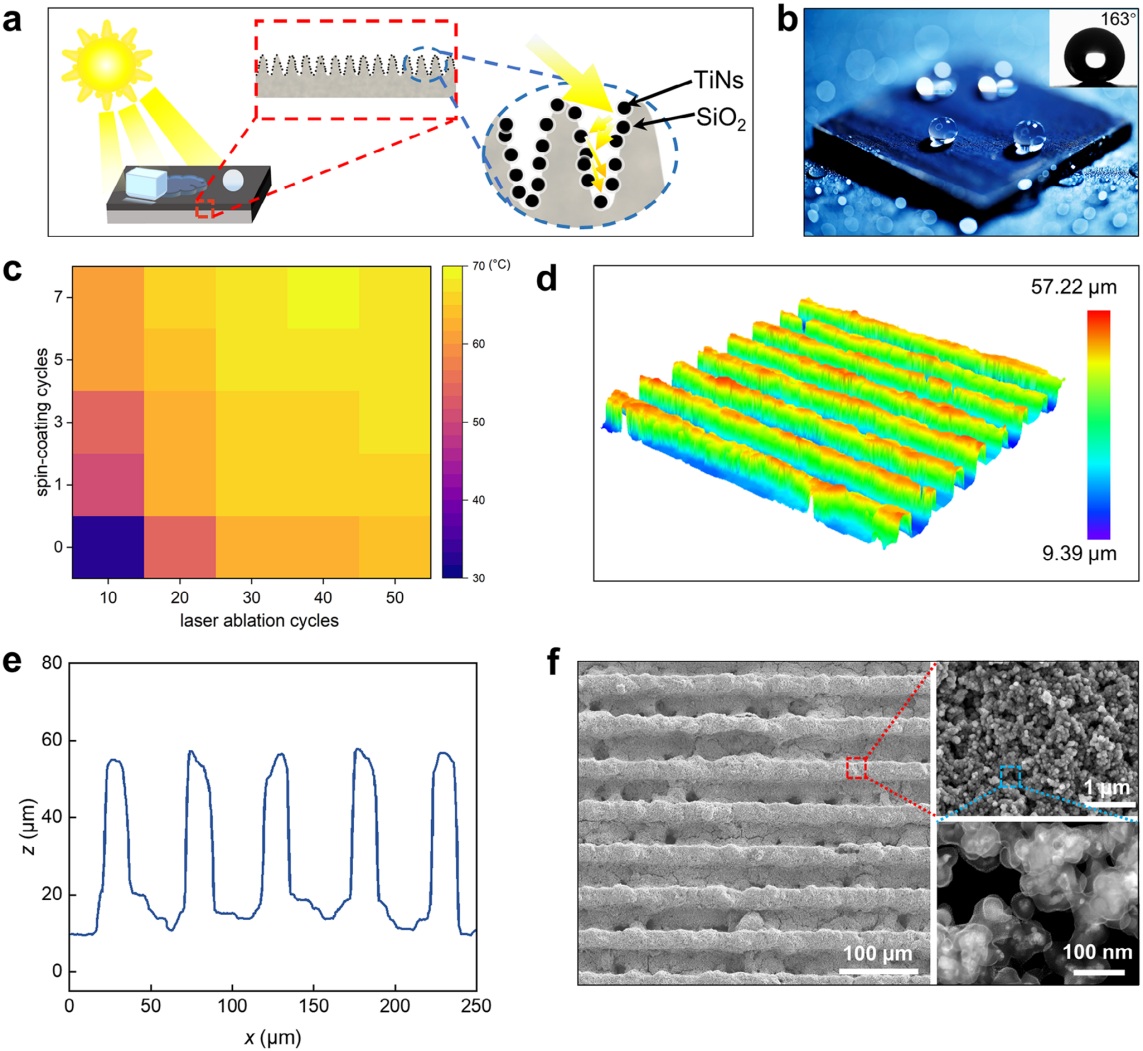
Design, mechanism, and characterization of the armored photothermal icephobic structured surface (APISS) coating. a) Schematic illustration of the APISS coating, highlighting its synergistic anti‐icing mechanism combining photothermal effects and superhydrophobicity. The composite micro‐nanostructure consists of laser‐ablated microgrooves, TiN nanoparticles, and a protective SiO_2_ shell. b) Optical image demonstrating the superior liquid repellency of the APISS coating on a stainless‐steel substrate. c) 2D color plot illustrating the relationship between laser ablation and spin‐coating cycles on temperature change for the APISS coating. Optimal processing parameters were determined to be 40 laser ablation cycles and 7 spin‐coating cycles d) 3D topographical profile of the microgroove array structure, revealing a regular pattern of parallel trenches. e) Cross‐sectional profile of the fabricated microgroove array, exhibiting an average depth of ≈45 µm. f) SEM image (left panel) showing the micro‐grooved structure with magnified section (upper‐right panel) revealing the nanostructured surface composed of TiN particles with average size of 20 nm and TEM image (lower‐right panel) demonstrating the core‐shell structure of TiN nanoparticles encapsulated by a 20±5 nm thick SiO_2_ protective layer.

As schematically illustrated in **Figure** **1**a, the APISS is engineered through a series of carefully optimized fabrication steps (Figure S1, Supporting Information) to incorporate laser‐ablated microgrooves, TiN nanoparticles, and a protective SiO_2_ shell. The fabricated APISS demonstrates exceptional liquid repellency, as evidenced by the high contact angle (≈160°) and low sliding angle (below 5°) of water droplets on the surface (Figure 1b; Movie S1, Supporting Information). To further illustrate the excellent self‐cleaning capability of the APISS structure, a dirt removal experiment was conducted using SiO_2_ microparticles (≈1 µm in diameter) as a simulated contaminant. Figure S2 (Supporting Information) clearly demonstrates that these microparticles deposited on the superhydrophobic APISS surface were easily captured and removed by rolling water droplets, confirming its superior self‐cleaning performance. To achieve the desired photothermal performance, we systematically investigated the number of spin‐coating cycles and laser ablation cycles (Figure S3, Supporting Information) to determine the optimal parameters. Our results revealed that in our configuration, 40 laser ablation cycles and 7 spin‐coating cycles imparted the best photothermal conversion efficiency with the highest temperature increase up to ≈70 °C under 1.5 suns solar illumination (Figure 1c). This optimization process highlights the importance of balancing the micro and nanostructures to maximize light absorption and photothermal conversion efficiency. Specifically, uniform parallel micro‐grooves with a depth of ≈45 µm, and pitch of ≈50 µm were achieved after 40 laser ablation cycles (Figure 1d,e). Scanning electron microscopy (SEM) imaging provides detailed visualization of the hierarchical structure, revealing TiN nanoparticles with an average size of 20 nm uniformly distributed across the surface (Figure 1f). The nanoparticles are well‐integrated into the microgrooved structure, creating a hierarchical network that contributes to both the superhydrophobic and photothermal properties of the coating. Transmission electron microscopy (TEM) confirms the core‐shell structure of the TiN nanoparticles encapsulated by a 20 ± 5 nm thick SiO_2_ protective layer, preventing the loss of nanoparticles and ensuring robustness against repeated icing‐deicing cycles. Energy‐dispersive X‐ray spectroscopy (EDS) revealed key elements contributing to the coating's functionality, including Si, Ti, and F, with a uniform spatial distribution ensuring consistent performance (Figure S4, Supporting Information).

### 2.2 Optical Properties and Photothermal Performance

**Figure 2:**
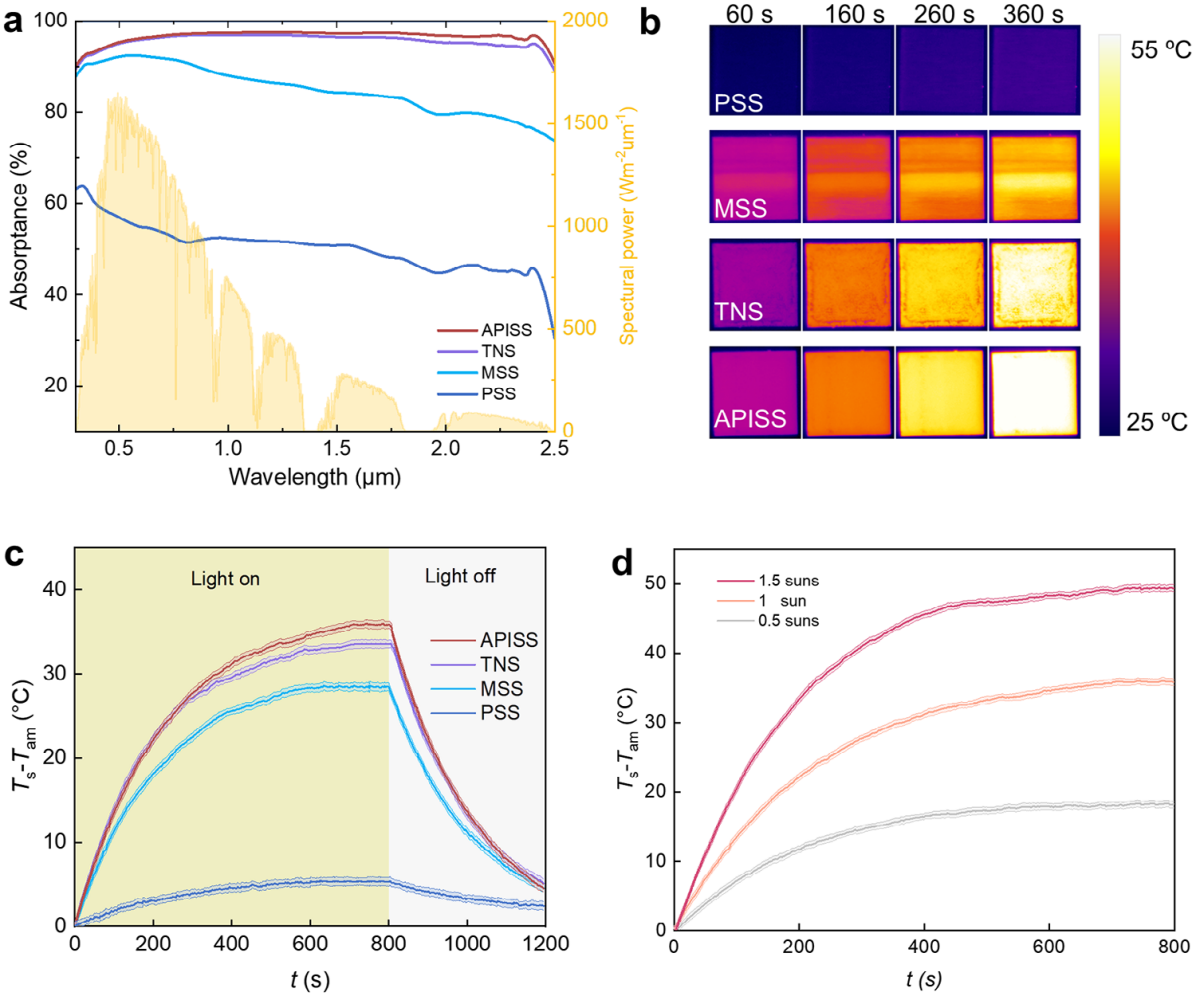
Optical properties and photothermal performance. a) Comparison of absorptance spectra for PSS, MSS, TNS, and APISS samples (left y‐axis). The yellow shaded area represents the AM 1.5 standard solar spectrum (right y‐axis). b) Time‐resolved infrared images showing temperature evolution of the four samples under 1 sun illumination at 60, 160, 260, and 360 s, respectively. c) Temperature change (*T*_s_‐*T*_am_) as a function of time under 1 sun illumination (light on) and subsequent cooling (light off). Shaded areas represent the s.d. from 10 independent measurements. d) Effect of light intensity (0.5, 1.0, and 1.5 suns) on the temperature increase performance of APISS coatings. Shaded areas represent the s.d. from 10 independent measurements.

To understand the exceptional anti‐icing performance of the APISS, we conducted a comprehensive analysis of its optical properties and photothermal performance. We compared the APISS to three types of control surfaces, i.e., pristine stainless steel (PSS), microstructured stainless steel without TiN (MSS), and TiN‐coated stainless steel without microstructures (TNS). SEM images of PSS, MSS, and TNS are shown in Figure S5 (Supporting Information). This comparison allows us to decouple the contributions of surface structures and TiN nanoparticles to the overall performance of the armored APISS. Specifically, the APISS coating demonstrated superior light absorption across the solar spectrum, particularly in the near‐infrared region which possesses a significant portion of solar energy (**Figure** **2**a), whereas the PSS coating shows the lowest absorptance across the spectrum, as expected for a reflective metal surface. The MSS surface demonstrates improved absorption compared to PSS, particularly in the visible range, due to light trapping effects of the microstructure. The TNS surface shows high absorption across the spectrum, highlighting the contribution of TiN nanoparticles to light absorption. We further calculated the average light absorption rate. $\bar{A}$ for each surface by using Equation (1):^[^ ^34^ ^]^

(1) $$\bar{A}=\frac{\int_{\lambda_{\min}}^{\lambda_{\max}}A(\lambda )d\lambda }{\lambda_{\max}-\lambda_{\min}}$$

where *A(λ)* represent absorptance at a wavelength *λ*, *λ*_max_ and *λ*_min_ equal to 2.5 and 0.3 µm, respectively. As a result, the absorption rates of APISS reaches 97.97%, 50.4%, 85.2%, and 95.94% higher than that of PSS, MSS, and TNS, respectively, further validating our above analysis.

To visualize the photothermal effect, we conducted time‐resolved infrared imaging of all samples under 1 sun illumination (Figure 2b). The images clearly show that the APISS achieves the highest temperature increase among all samples. Quantitative analysis of the temperature difference between the sample surface *T*_s_ and the ambient environment *T*_am_ over time provides further insights into the photothermal performance of each surface (Figure 2c). The APISS coating demonstrates a rapid temperature increase, reaching a Δ*T* = *T*_s_ – *T*_am_ of ≈35 °C after illumination for 800 s. This result significantly outperforms the other surfaces, with TNS showing Δ*T* of ≈33 °C, followed by MSS (Δ*T* ≈ 28 °C), and finally PSS with negligible temperature increase (Δ*T* < 5 °C). The coating also exhibited consistent performance under varying light intensities, achieving Δ*T* values of 18, 35 °C, and 49 °C under 0.5, 1, and 1.5 suns, respectively (Figure 2d), which is particularly important for real‐world applications where solar illumination can vary due to factors such as time of day, season, and atmospheric conditions.

### 2.3 Anti‐Icing and Deicing Performance

**Figure 3:**
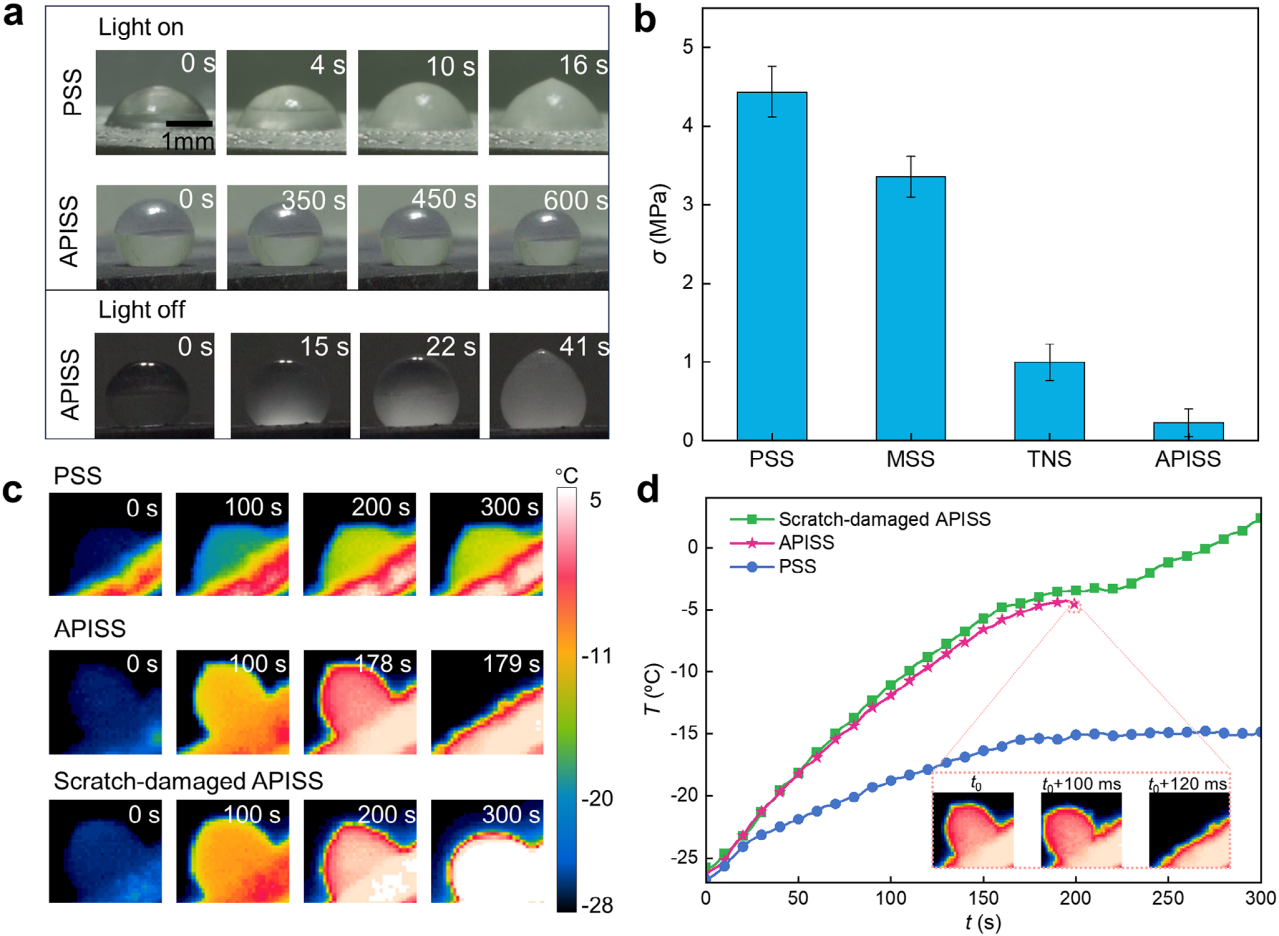
Anti‐icing and deicing performance of APISS for single‐droplet experiments. a) In situ freezing process of a 10 µL water droplet on APISS surface under light on (top row) and light off (bottom row) conditions. The chamber temperature was controlled at −25 °C with 65% relative humidity. Time stamps indicate the progression of the freezing process. b) Comparison of adhesive force for a 10 µL droplet on PSS, MSS, TNS, and APISS surfaces without illumination (light off). c) Infrared imaging comparison of the melting process for a frozen 5 µL droplet on 30° tilted PSS, APISS, and scratch‐damaged APISS surfaces under 1 sun illumination. The color scale represents temperature from −27 to 3 °C. d) Mean temperature (Td) variation of the frozen droplet as a function of time during de‐icing under 1 sun illumination for PSS, APISS, and scratch‐damaged APISS. The inset shows detailed dynamics of droplet detachment on APISS surface, with t0 indicating the initial period of 178s before rapid melting begins.

The anti‐icing performance of the APISS was evaluated by observing a 10 µL droplet freezing behavior in environment with temperature −25 °C and relative humidity of ≈65% under 1 sun intensity illumination. As shown in the upper panel of **Figure** **3**a, on the untreated PSS substrate, the droplet froze within 16 s due to its relatively weak photothermal effect. In contrast, on the APISS, it was clearly observed that the droplets remained unfrozen throughout the observation period. The prolonged freezing behavior of droplets on APISS samples can be rationalized from the perspective of thermodynamic analysis, which reveals that the related heat loss of the droplet on APISS is much smaller than that on control samples (Note S1, Supporting Information). Moreover, by comparing the droplets’ profiles at time 0 s and 600 s, we noted an obvious trend of droplet volume decreasing, indicating the partial evaporation of droplet. Subsequently, to individually validate the APISS's superior structure effect on anti‐icing performance, we conducted further tests on the sample surface under conditions without illumination, keeping all other conditions unchanged (Figure 3a, lower panel). We observed that, within 15 s, the droplets on the APISS surface instantly became a translucent state as ice began to form around the droplet's perimeter. Inside the droplet, freezing started from the bottom layer and gradually spread upward over time, until a tip formed at the droplet's top at 41 s. The experimental results of the other three types of surfaces, PSS, MSS, and TNS, under non‐illumination conditions, are shown in Figure S6 (Supporting Information). Without illumination, the droplet on the APISS surface exhibited the longest freezing time among all surfaces, demonstrating its superior anti‐icing performance. In addition, for the scenario involving droplet dynamic impact behavior, the APISS coating still demonstrates superior anti‐icing performance with instant droplet bouncing and removal from the substrate (Figure S7, Supporting Information). Moreover, the ice adhesion strength was measured using a customized setup (Figure S8, Supporting Information), following a similar characterization method reported previously.^[^ ^43^ ^]^ The ice adhesion strength results showed that the APISS coating exhibited the lowest adhesion strength (≈0.2 MPa) compared to PSS (≈4.4 MPa), MSS (≈3.3 MPa), and TNS (≈1 MPa) (Figure 3b). The exceptionally low ice‐adhesion strength of our APISS sample (≈0.2 MPa) is also comparable to state‐of‐the‐art superhydrophobic surfaces previously reported in literature.^[^ ^23^, ^44^ ^]^

Moreover, the deicing performance of the APISS coating was assessed through experiments designed to mimic real‐world conditions. After the water droplets of 10 µL were frozen on sample surfaces with temperature and environmental humidity of −25 °C and 65%, respectively, the melting and detachment processes from 30° tilted surfaces were observed using infrared imaging under 1 sun illumination (Figure 3c). Specifically, On the PSS surface, the frozen droplet showed minimal change throughout the experiment, with only a slight increase in temperature but no detachment. In contrast, the APISS demonstrated rapid and efficient deicing. The frozen droplet on the APISS coating started to melt at the solid‐liquid interface within 178 s of illumination. Complete detachment of the droplet occurred at 179 s, with the droplet sliding off the tilted surface due to gravity (Movie S2, Supporting Information). A visible‐light imaging comparison of the melting process on different surfaces is also provided in Figure S9 (Supporting Information), further supporting the infrared imaging results.

Interestingly, even a deliberately scratch‐damaged APISS surface showed improved deicing performance compared to PSS. The scratch‐damaged APISS was prepared by scratching the coating surface using a sharp knife so that the water droplet could easily get pinned on the damaged area (Figure S10, Supporting Information). While the detachment process was much slower than on the intact APISS surface, the scratch‐damaged coating still facilitated melting and provided the possibility for eventual removal using external shear forces such as gravity. This observation underscores the resilience of the APISS coating and suggests that it may maintain functionality even after sustaining some damage. Quantitative analysis of the mean temperature (*T*_d_) of the frozen droplets during the deicing process provides further insights into the performance of each surface (Figure 3d). On the PSS surface, the droplet temperature increases slowly and plateaus ≈−16 °C, insufficient for melting. The APISS surface, however, shows a rapid increase in *T*_d_, reaching ≈5 °C at time *t*_0_ = 178 s. Though the measured *T*_d_ is slightly lower than the melting point, the actual temperature near the droplet bottom has significantly increased above the measured *T*_d_ due to the superior photothermal effect from the underlying APISS, leading to fast melting of the droplet near the contact line area. The formed thin liquid layer makes the droplet stay in Cassie state, resulting in fast detachment within only ≈120 ms (Inset of Figure 3d). Meanwhile, the scratch‐damaged APISS demonstrates an intermediate performance, with the measured *T*_d_ continuously increasing to above melting point. Despite adhesion due to the contact line pinning caused by damage of the surface, the underlying photothermal properties continue to contribute to ice melting. Compared to PSS coating, our proposed APISS also demonstrates excellent large ice sheet removal capability (Figure S11, Supporting Information).

### 2.4 Durability and Robustness Tests

**Figure 4:**
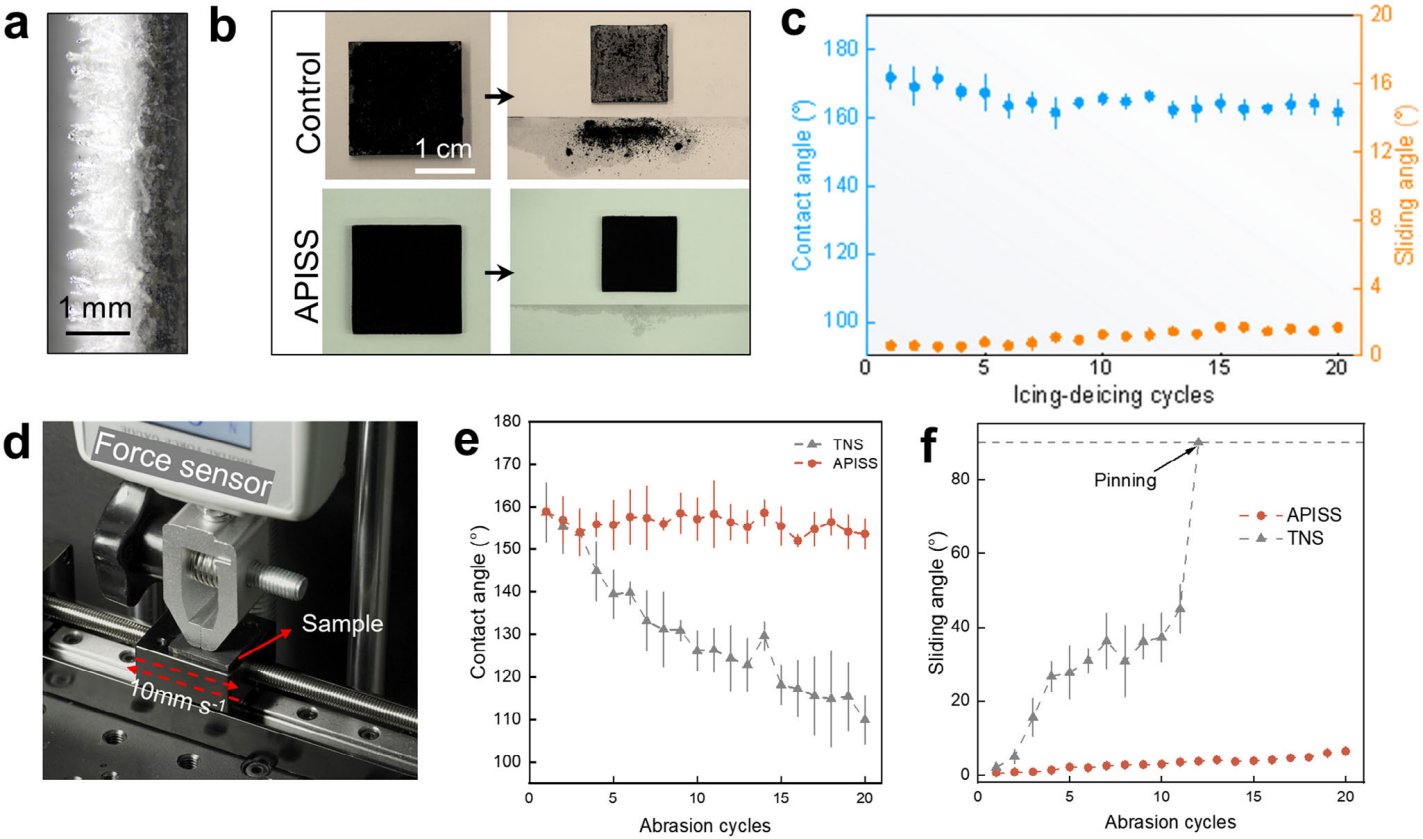
Durability and robustness of the APISS coating. a) Side‐view image of ice formation on the APISS surface, showing controlled ice growth to ≈1 mm thickness. b) Comparative analysis of particle retention after icing‐deicing cycles on control (top) and APISS (bottom) coatings. The control surface shows significant TiN nanoparticle shedding onto the underlying surface, while the APISS coating exhibits minimal particle loss. c) Superhydrophobicity retention of the APISS coating over 20 icing‐deicing cycles, demonstrating consistent contact angles (color blue) above 160° and low sliding angles (color orange) below 2°. Error bars represent s.d. from 10 independent measurements. d) Photograph showing the linear abrasion setup with a normal load of ≈14 kPa. (e,f) The measured contact angle e) and sliding angle f) variations over 20 consecutive abrasion cycles. Despite a slight change for APISS coating, the contact angle remains above 150° and the sliding angle remains below 6° after 20 cycles, indicating excellent mechanical durability compared to the TNS sample. Error bars represent s.d. from 10 independent measurements.

We assessed the coating's durability through rigorous icing‐deicing cycles and mechanical abrasion tests. In icing‐deicing experiments, we compared the performance of the APISS coating to a control surface without the SiO_2_ protective shell. Ice formation was carefully controlled to achieve a thickness of ≈1 mm on both surfaces under the conditions of temperature −25 °C, relative humidity (RH) 65%, and standard atmospheric pressure (**Figure** **4**a). Interestingly, the control surface showed substantial shedding of TiN nanoparticles, as evidenced by the accumulation of black particles on the underlying surface after the melting of the ice (Figure 4b upper panel and Movie S3, Supporting Information). This particle loss behavior is detrimental to the long‐term photothermal performance of the coating. In contrast, the APISS coating exhibited minimal particle loss under the same conditions (Figure 4b lower panel and Movie S4, Supporting Information). The SiO_2_ protective shell effectively “armored” the TiN nanoparticles, preventing their detachment during the harsh icing‐deicing process. To quantify the coating's ability to maintain its superhydrophobic properties over repeated icing‐deicing cycles, we measured the variations of both contact angle and sliding angle for a total of 20 cycles (Figure 4c). Remarkably, the superhydrophobicity of the APISS coating remained highly consistent throughout the experiment. The contact angle stayed above 160°, and similarly, the sliding angle remained below 2° throughout the testing after 20 cycles. These results demonstrate the excellent durability of the APISS coating's superhydrophobic properties under repeated icing‐deicing conditions.

Specifically, the abrasion was performed using an aluminum alloy probe as the indenter with a defined vertical pressure (≈14 kPa) and reciprocating linear abrasion with wear distance of 20 mm for each cycle (Figure 4d). This process was repeated for 20 cycles to assess the long‐term mechanical resilience of the coating. Briefly, the as‐proposed APISS demonstrated negligible liquid repellency deterioration, which can still maintain a high contact angle (Figure 4e) and low sliding angle (Figure 4f) compared to TNS. The long‐term durability of APISS is ascribed to the existence of large‐scale microstructure that houses mechanically fragile TiN nanoparticles, thus preventing the removal of the nanostructures by abrasions (Figure S12, Supporting Information). The contact angle of the APISS coating remained above 150°, and the sliding angle, while showing a more noticeable increase, still remained below 6°, indicating that the APISS coating retains its superhydrophobic characteristics even under severe mechanical wear. To more intuitively demonstrate the durability of the APISS's superhydrophobicity, we also calculated the contact angle recovery rate^[^ ^23^ ^]^ of droplets on the surface *δ*_a_ that can be expressed by *δ*_a_ = 1‐ (*θ*_0_ – *θ*_f_)/*θ*_0_, where *θ*_0_ represent the initial contact angle of the droplet before freezing, and *θ*_f_ denote the contact angle of the droplet after ice‐melting cycles. After 20 ice‐melting cycles, the contact angle recovery rate *δ*_a_ is ≈94%. Similarly, after 20 mechanical wear tests, the surface also achieves a high contact angle recovery ratio of ≈92%. We also conducted high‐speed jet impact tests to verify the durability of APISS (Figure S13, Supporting Information). The robust performance of the APISS under icing‐deicing cycles, mechanical abrasion tests, and high‐speed jet impact tests can be attributed to several factors. The hierarchical micro‐nanostructure provides a stable foundation for the coating, while the SiO_2_ shell effectively protects the TiN nanoparticles from detachment. Additionally, the fluorination treatment penetrates the surface structure, ensuring that the low surface energy is maintained even as the outermost layer experiences wear.

### 2.5 Defrosting and Anti‐Frosting Performance

**Figure 5:**
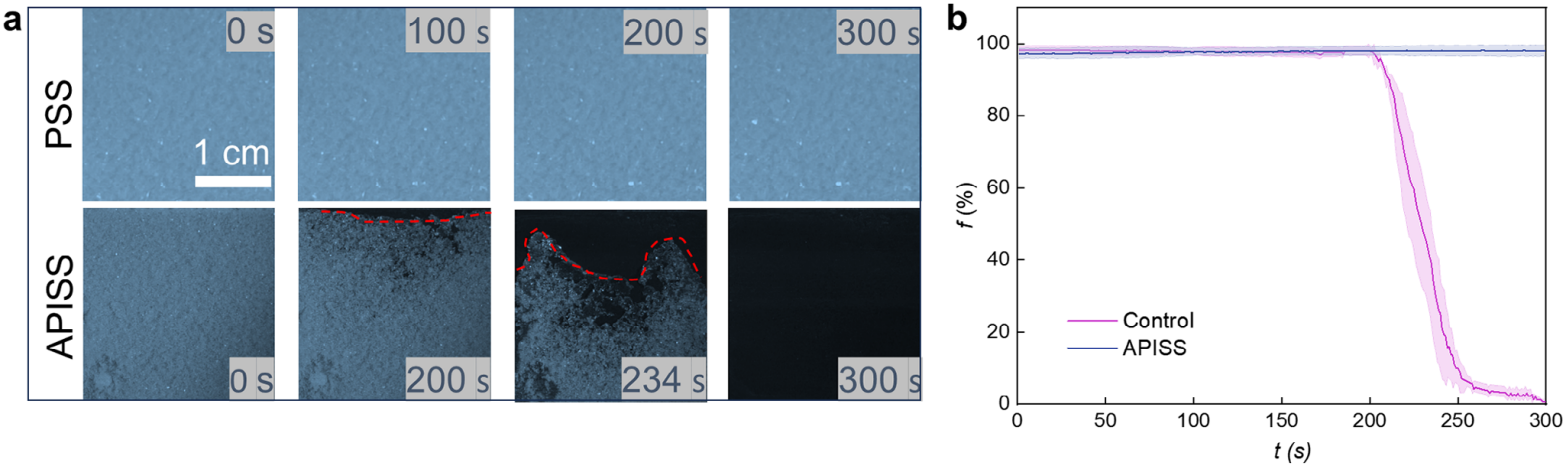
Defrosting performance comparison between APISS and control (PSS) surfaces. a) Time‐resolved defrosting process under 1 sun illumination with substrate temperature maintained at −25 °C. The top row shows the control (PSS) surface remaining frosted throughout the experiment. The bottom row demonstrates the APISS surface, where frost gradually melts and completely disappears within 300 s. Red dashed lines highlight the frost‐free areas on the APISS surface. b) Frost fraction *f* as a function of time for both APISS and control samples. APISS demonstrates superior defrosting potential with a rapid decrease in frost coverage, while the control sample maintains nearly 100% frost coverage throughout the experiment. Shaded areas represent the standard deviation from 10 independent measurements.

The ability to remove frost efficiently is crucial for many real‐world applications. We further evaluated the defrosting capabilities of our APISS coating by comparing its performance to a controlled PSS surface under −25 °C with a relative humidity of 65%. As shown in **Figure** **5**a, ≈28% of the frost had been removed from the APISS within ≈234 s of illumination, and complete defrosting (>95% frost removal) was achieved in less than 300 s, demonstrating its remarkable defrosting efficiency. In striking contrast, the PSS surface showed minimal change throughout the experiment, retaining nearly complete frost coverage even after 300 s of illumination. To further quantify the defrosting dynamics, we analyzed the variation of frost fraction *f*, i.e., the proportion of the surface covered by frost, as a function of time (Figure 5b). The APISS exhibited a rapid decrease in frost fraction, following a sigmoidal curve characteristic of efficient defrosting processes. Conversely, the PSS surface maintained a nearly constant frost fraction (*f* ≈ 1) throughout the experiment, indicating negligible defrosting. We defined the defrosting time *t*_de_ as the duration required to reduce the frost coverage to less than 5% (*f* < 0.05). For the APISS, *t*_de_ was ≈300 s under the given conditions. The PSS surface did not achieve significant defrosting within the experimental timeframe.

In addition to its active frost removal capabilities, our APISS also demonstrates robust passive frost resistance under harsh conditions (−25 °C, RH ≈ 65%). As shown in Figure S14 (Supporting Information), the APISS coating significantly delays frost formation compared to the PSS surface without illumination. After 200 s, the frost coverage on APISS remains below 50%, while PSS is completely frosted. The exceptional anti‐frosting performance of APISS can be attributed to the synergistic effect of its superhydrophobicity. Microscopic observations reveal that condensate water droplets on the APISS exhibit self‐jumping behavior due to coalescence, effectively removing potential frost nucleation sites (Figure S15 and Movie S5, Supporting Information). This self‐cleaning mechanism, combined with the localized heating from the photothermal effect, creates an environment highly resistant to frost formation.

## 3 Conclusion

In conclusion, by seamlessly integrating superhydrophobicity with photothermal effects, APISS offers a robust, efficient, and sustainable solution for mitigating icing across diverse applications. The hierarchical micro‐nanostructures and TiN nanoparticle integration deliver exceptional photothermal performance and durability under harsh environmental conditions. Under 1 sun illumination, APISS achieves a rapid temperature increase of 35 °C and melts ice within 179 s, significantly outperforming conventional surfaces. Its superhydrophobicity, characterized by a water contact angle >160° and sliding angle <5°, ensures the effective removal of melted water and prevents refreezing. Rigorous testing confirms its resilience over 20 icing‐deicing cycles, maintaining 94% superhydrophobic performance and high durability under abrasion. Unlike traditional methods, APISS addresses critical limitations such as high energy consumption, environmental impact, and scalability, making it an ideal candidate for applications ranging from renewable energy systems and aviation to infrastructure in cold climates. This study establishes APISS as a benchmark for next‐generation anti‐icing materials, offering a pathway toward broader adoption of sustainable and multifunctional technologies in real‐world settings.

## 4 Experimental Section

### Chemicals and Materials

Trichloro (1H,1H,2H,2H‐tridecafluoro‐n‐octyl) silane (97%), Ethanol (99.8%), Ammonia solution (28%), and Titanium nitride (20 nm) was obtained from Shanghai Aladdin Biochemical Technology Co., Ltd. The tetraethyl orthosilicate (TEOS) (99%), acetone, and hexane were acquired from Sigma–Aldrich. Hydrochloric acid (36%) was purchased from Dongjiang Chemical Reagent Co., Ltd. All chemicals were used without further purification.

### Surface Fabrication

The mechanically polished stainless‐steel sheets with size of 20 mm × 20 mm × 2 mm were first cleaned ultrasonically in acetone, ethanol, and deionized water, respectively, and then dried in an 60 °C oven. For the fabrication of APISS, the cleaned sample substrates were processed using a 10 W UV laser marking machine (KY‐M‐UV10J, Wuhan Keyi Co., Ltd.) with a repetition rate of 40 kHz and central wavelength of 355 nm to create the periodic rough microstructures. The entire ablation process was repeated 40 times to ensure the desired structure etching depth. To improve adhesion and create a uniform coating of the TiN nanoparticles, all samples were immersed in a 5 m HCl solution for 1 h, ultrasonically cleaned in ethanol and water, and then placed on a hot plate at 500 °C for 4 h for oxidation treatment. TiN nanoparticles dispersed in ethanol with a 0.2% (volume) concentration were spin‐coated on all sample surfaces using a spin coater (KW‐4B, Beijing Setcas Ltd.), followed by drying at 80 °C for 30 min. A SiO_2_ shell was deposited on sample surfaces via CVD using TEOS and ammonia under vacuum conditions (−0.8 kPa) for 24 h. Lastly, all types of samples were immersed in 1 mm trichloro(1H,1H,2H,2H‐perfluorooctyl) silane/hexane solution for 2 h, followed by rinsing with ethanol and heating at 100 °C for 1 h to render the sample surfaces superhydrophobic.

### Surface Characterizations

Laser‐etched micro‐groove‐like sample surfaces were examined using an ultra‐depth‐of‐field microscope (DSX1000, Olympus). The micro‐nanoscale morphology and elemental composition of surface were examined by a field emission scanning electron microscope (Zeiss Supra 55VP) equipped with an energy‐dispersive spectroscope. The samples used for TEM observation were prepared using a focused ion beam scanning electron microscope (Crossbeam 350, Zeiss), and the core‐shell structure of TiN nanoparticles was confirmed by a transmission electron microscope (JEM‐3200FS, JEOL). Contact angles and sliding angles were measured using a contact angle goniometer (OCA25, DataPhysics Instruments). The reflectance spectra of all samples across the wavelength range of 300–2500 nm were collected using a UV–vis–NIR spectrometer (Lambda 1050, Perkin Elmer), equipped with an integrating sphere detector. The absorptance rate was calculated as 100% – reflectance, assuming negligible transmission due to the thick substrates.

### Experiment Setup

A custom experimental setup consisted of a hollow circulating chamber, polystyrene foam, and a supercooled liquid system. The polystyrene foam was adhered to the exterior of the chamber, with additional foam placed beneath all tested samples. The circulating liquid inside the chamber was supercooled ethanol, supplied by a low‐temperature thermostatic bath (LC‐LTC‐5/40, Lichen Ltd.). A LED lamp was used to simulate sunlight exposure, whose light intensity was adjusted using a photometer. The T‐type thermocouple was used to measure both the ambient temperature in the chamber and the temperature of all samples.

For the anti‐icing performance test, the experiment was conducted in the environment with the temperature set to −25 °C, and 10 µL droplets generated by a syringe Pump (LSP01‐3A, Langer Instruments Ltd.) were precisely dispensed onto all substrates mounted by a sample holder. The freezing behavior of droplets was monitored by a digital camera. In the de‐icing experiment, the sample holder was inclined by an angle of 30° after the complete freezing of droplets, and the LED lamp was tailored to generate 1 sun intensity. The continuous temperature change of the droplets and the melting process were recorded by an infrared camera (A615, FLIR)

For the anti‐frosting performance evaluation, all the tested samples were placed on a Peltier cooling stage with temperature set at −25 °C in the ambient environment (RH = 65%). During the freezing process, the coalescence‐induced droplet bouncing behavior on the sample surface was captured by a digital camera equipped with a microscope lens (Navitar 12× zoom lens). For the de‐frosting experiments, the frost‐coated samples were then removed from the Peltier cooling stage and placed inside the cooling chamber with air temperature maintained at −25 °C. Then, the LED lamp was turned on and the defrosting behavior of the test samples was recorded using a digital camera.

### Ice Adhesion Strength Test

The ice adhesion strength of frozen droplets on various samples was measured via a digital force gauge with accuracy up to 0.01 N (SC‐50N, Shence Ltd.), which was connected to a computer to record the force variation during the pushing operation until the frozen droplets were fully detached from the surfaces. Then the ice adhesion strength was calculated based on the measured peak force and droplet/surface contact area.

## Conflict of Interest

The authors declare no conflict of interest.

## Supporting information



Supporting Information

Supplemental Movie 1

Supplemental Movie 2

Supplemental Movie 3

Supplemental Movie 4

Supplemental Movie 5

## Data Availability

The data that support the findings of this study are available from the corresponding author upon reasonable request.
