# Supplementary material for: Hierarchical Icephobic Surfaces with Enhanced Photothermal Performance for Sustainable Anti‐Icing
Source: Adv Sci (Weinh). 2025 May 8;12(27):2502945. doi: 10.1002/advs.202502945 (PMC12279169; doi:10.1002/advs.202502945)
Supplement: Supplementary file 1 — Supporting Information [file ADVS-12-2502945-s006.docx]

Supporting Information

**Hierarchical icephobic surfaces with enhanced photothermal performance for sustainable anti-icing**

*Lei Zhang, Yongle Feng, Xixin Cao, Yue Dong, Weihong Liu, Bing Li, Jing Li^*^, Chonglei Hao^*^*

**This file includes**

Supplementary Note

Supplementary Figures S1 to S15

Other Supplementary Material for this manuscript includes the following:

Supplementary Movies S1 to S5

**Supporting Note-Thermodynamic analysis for anti-icing**

From the perspective of thermodynamic analysis, the delayed freezing time $\Delta t$ of a droplet on a supercooled surface can be expressed by using the equation^[1]^:

$$\Delta t=\frac{\rho\Omega c(T_{\text{am}}-T_{\text{s}})+\rho\Omega l}{\Delta q}$$

Where *ρ*, Ω, *c*, and *l* represent the droplet's density, volume, heat capacity, and latent heat, respectively; *T*_am_ and *T*_s_ represent the temperature of the ambient and the supercooled surface, respectively; Δ*q* denotes the net heat loss of the droplet on the supercooled surface per unit time. Theoretically, Δ*q* can be explained by the heat transfer between the droplet and the supercooled surface $\Delta q=q_{\text{l}}-q_{\text{g}}$, where $q_{\text{l}}$ and $q_{\text{g}}$ represent the heat lost and gained by the droplet, respectively. As the surface temperature drops, Δ*q* > 0. For a droplet deposited on our PSS sample, the net heat loss can be expressed by $\Delta q_{\text{P}}=q_{\text{l}}-q_{\text{g}}$. For the micro-nano structured superhydrophobic surface, due to the existence of the air cushion layer between the supercooled surface and the droplet, the net heat loss needs to consider the heat loss at the solid-gas interface and the solid-liquid interface, which can be expressed by the following formula^[2]^:

$$\Delta q'=q_{\text{ls}}+q_{\text{la}}-q_{\text{g}}$$

where $q_{\text{ls}}$ and $q_{\text{la}}$ respectively represent the heat loss at the solid-gas interface and the air-liquid interface. Considering that under illumination, the additional heat generated by light will be absorbed by both the supercooled surface and the droplet, the net heat loss ($\Delta q''$) for a superhydrophobic surface without photothermal effects can be expressed as:

$$\Delta q''=q_{\text{ls}}+q_{\text{la}}-q_{\text{g}}-q_{\text{s}}$$

where $q_{\text{s}}$ represents the heat introduced by light. The fabricated APISS coating, with exceptional photothermal effect, whose net heat loss in low temperature condition can be expressed as:

$$\Delta q_{\text{A}}'''=q_{\text{ls}}+q_{\text{la}}-q_{\text{g}}-q_{\text{pt}}-q_{\text{s}}$$

where $q_{\text{pt}}$ represents the heat obtained by the surface photothermal effect. In summary, $\Delta q_{\text{A}}'''$ of APISS is relatively smaller under the same conditions compared to Δ*q*_p_ for PSS sample, which consequently increases the time Δ*t* for droplet freezing. Therefore, the APISS coating exhibits superior anti-icing performance with much longer delayed freezing time.

**Supplementary Figures**


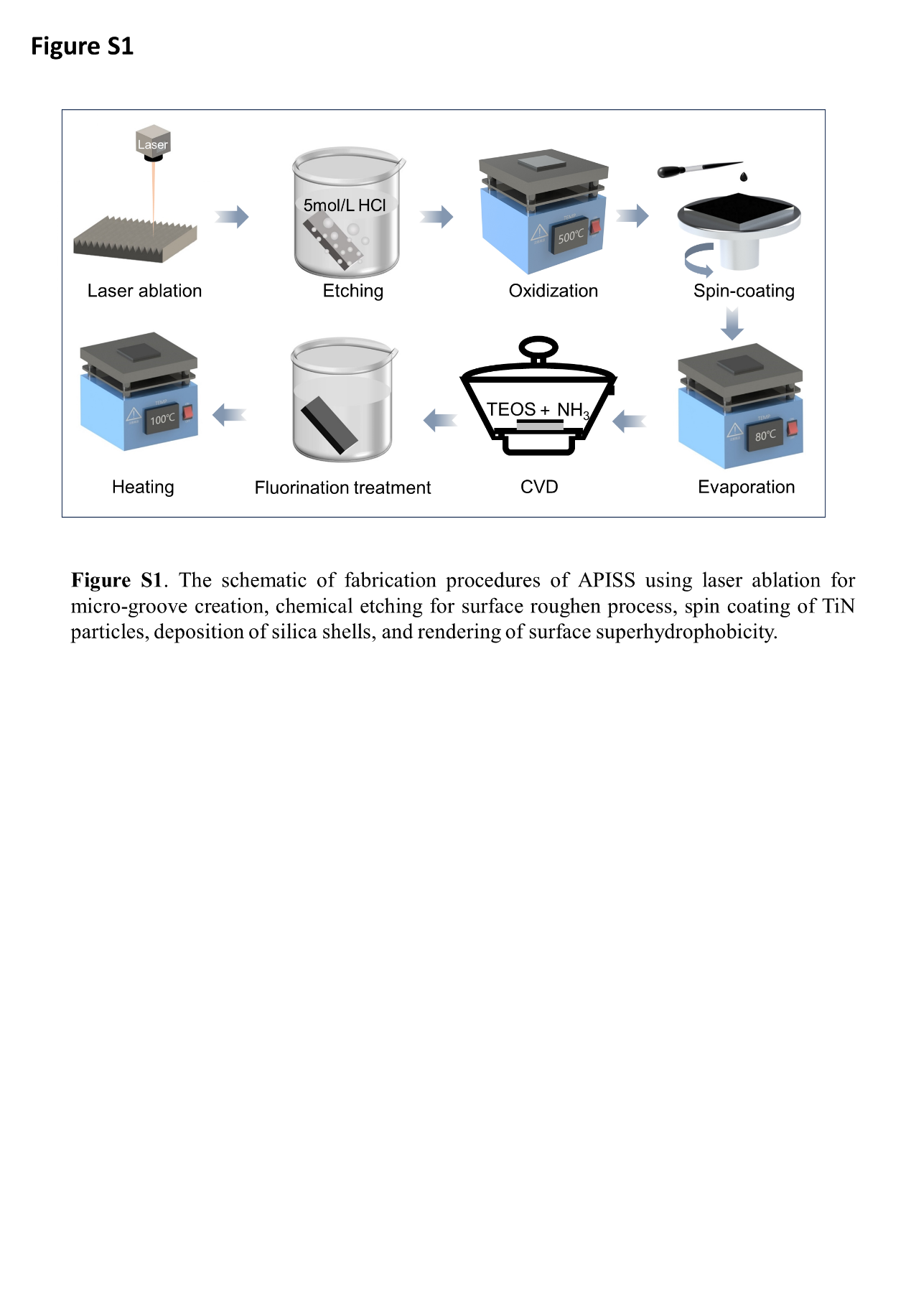


Supplementary Figure S1. The schematic of fabrication procedures of APISS using laser ablation for micro-groove creation, chemical etching for surface roughen process, spin coating of TiN particles, deposition of silica shells, and rendering of surface superhydrophobicity.


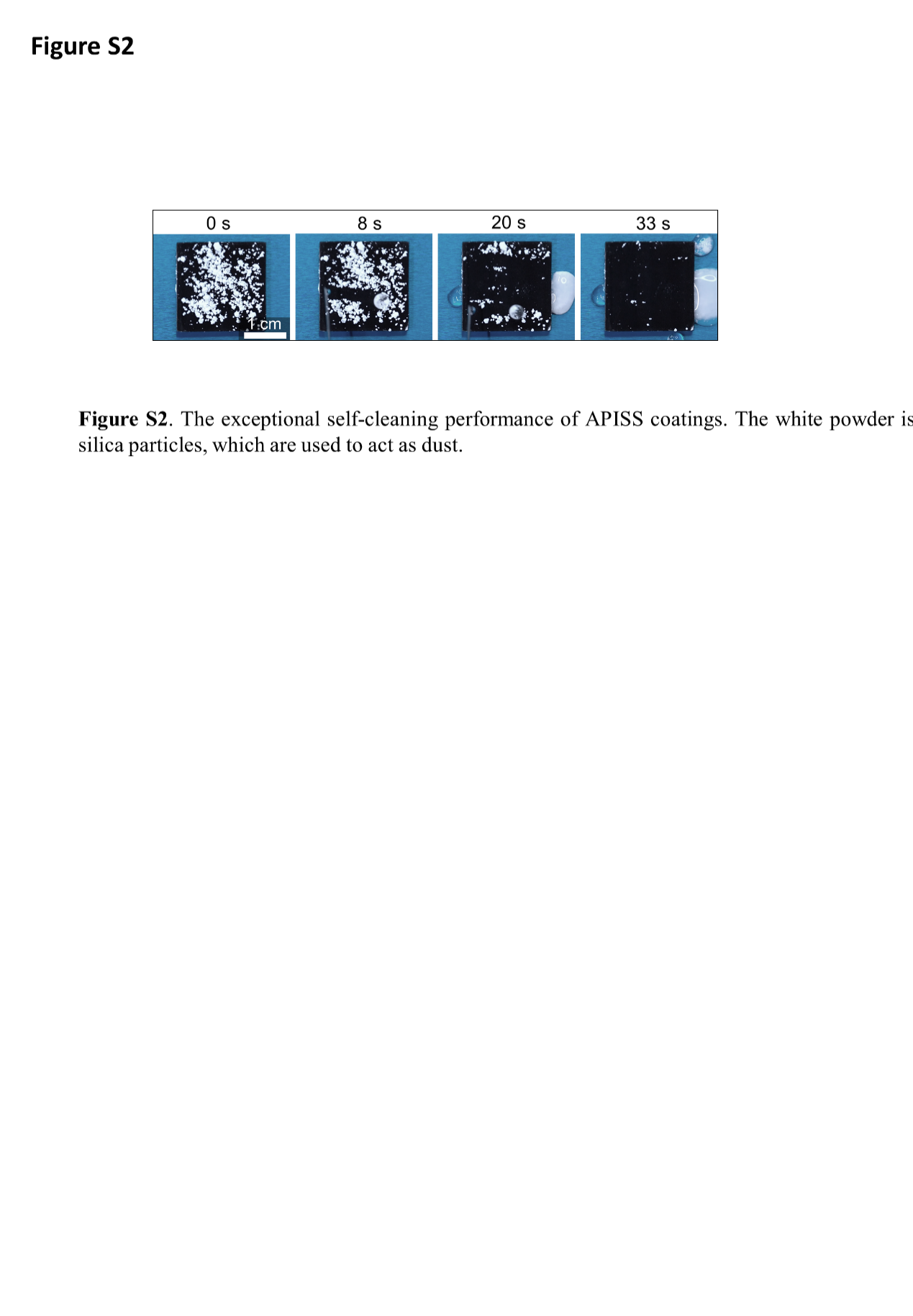


Supplementary Figure S2. The exceptional self-cleaning performance of APISS coatings. The white powder is silica particles (~1 μm in diameter), which are used to act as dust.


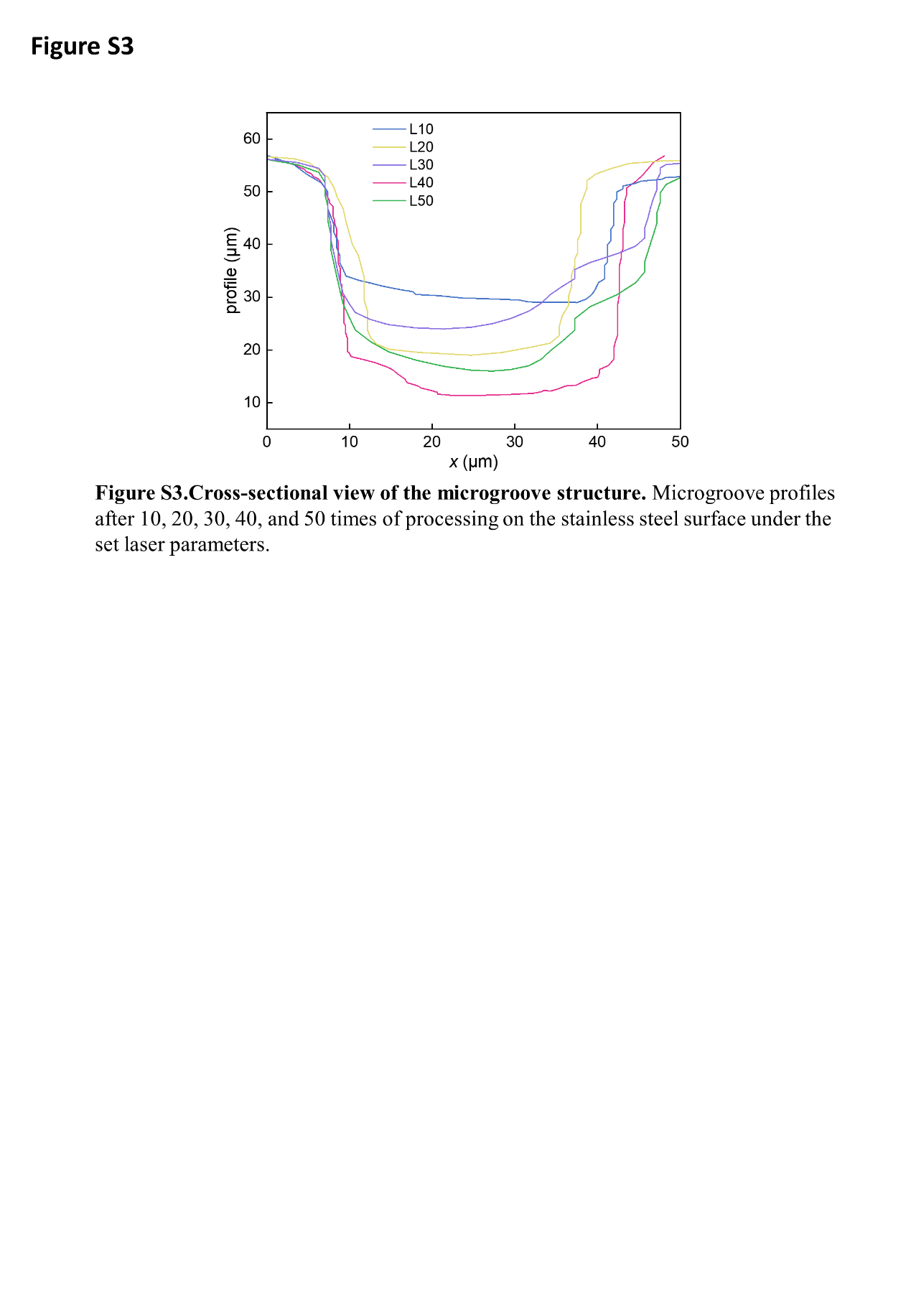


Supplementary Figure S3. The comparison of the cross-sectional profiles of the microgroove structure with different laser ablation cycles.


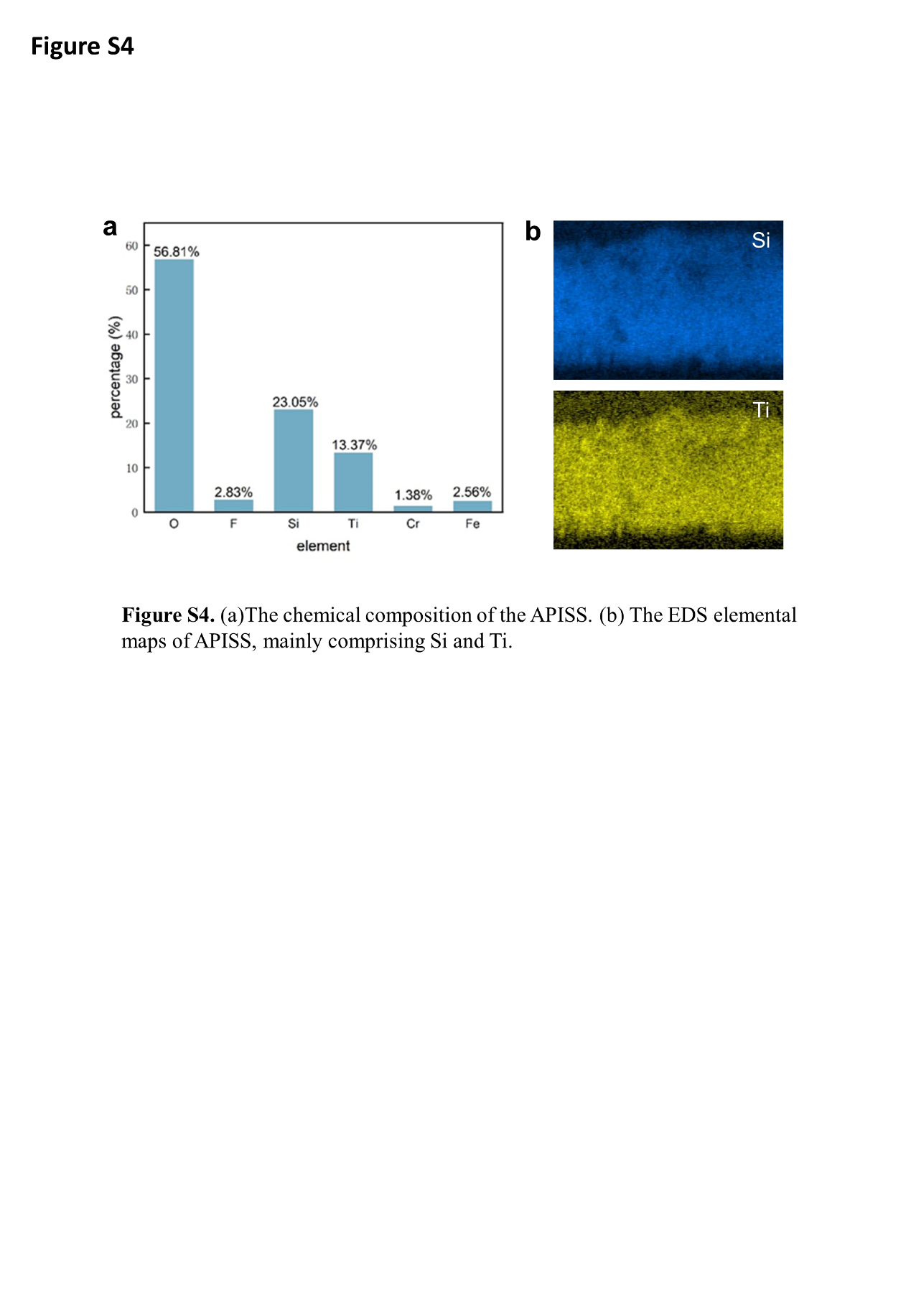


Supplementary Figure S4. Elemental composition and mapping analyses of the APISS coating. (a) Elemental composition analysis of the APISS coating, as determined by energy-dispersive X-ray spectroscopy (EDS). The chart illustrates the atomic percentage of key elements, with oxygen (O) comprising 56.81%, silicon (Si) 23.05%, titanium (Ti) 13.37%, fluorine (F) 2.83%, chromium (Cr) 1.38%, and iron (Fe) 2.56%. (b) Elemental mapping of the coating visualizing the uniform spatial distribution of Si) and Ti, demonstrating the structural uniformity and compositional integrity of the APISS surface.


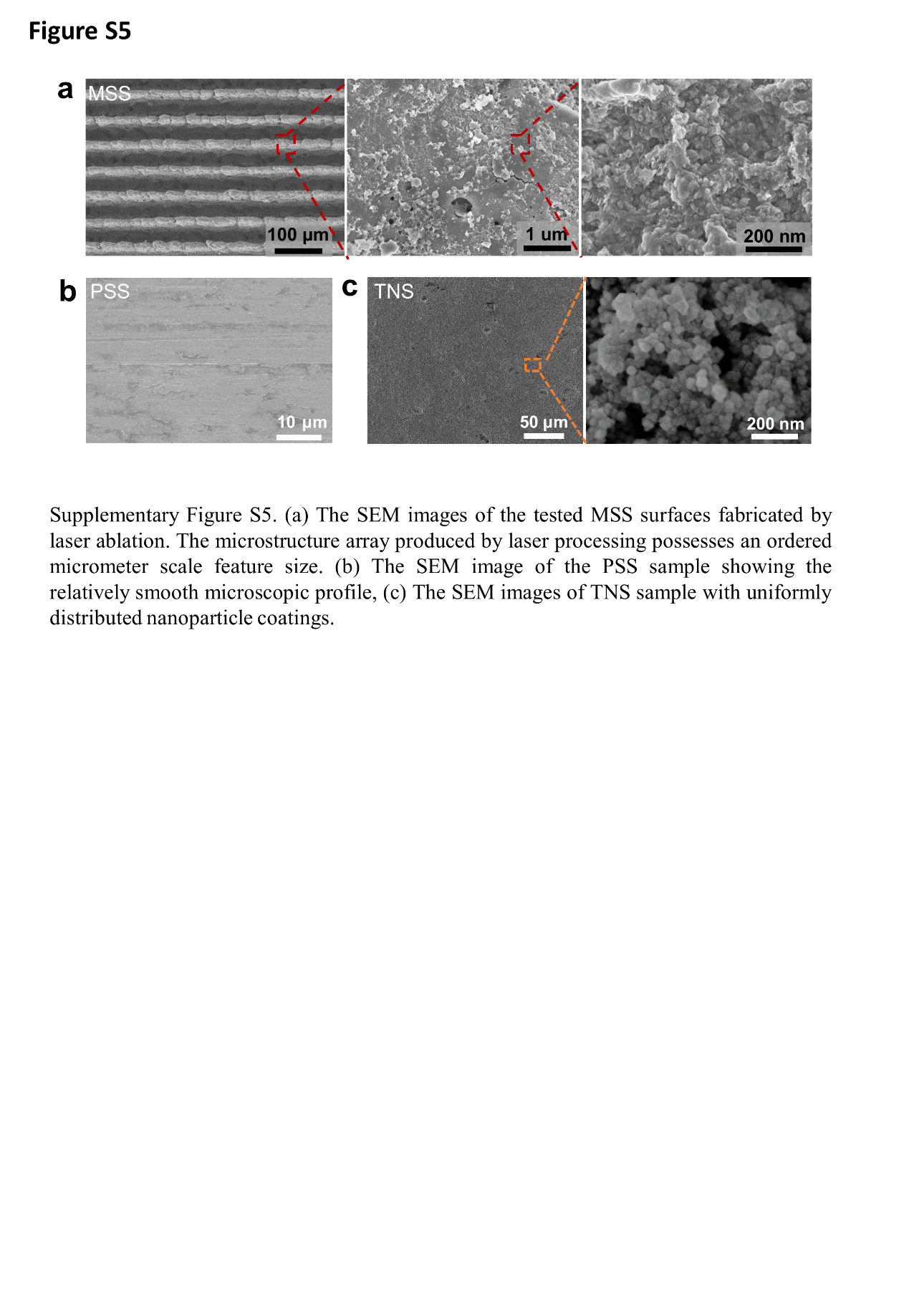


Supplementary Figure S5. (a) The SEM images of the tested MSS surfaces fabricated by laser ablation process, showing well-defined microscale groove structures. (b) The SEM image of the PSS sample showing a relatively smooth surface without micro- or nanostructures. (c) The SEM images of the TNS sample, exhibiting a uniform nanoparticle coating across the surface.


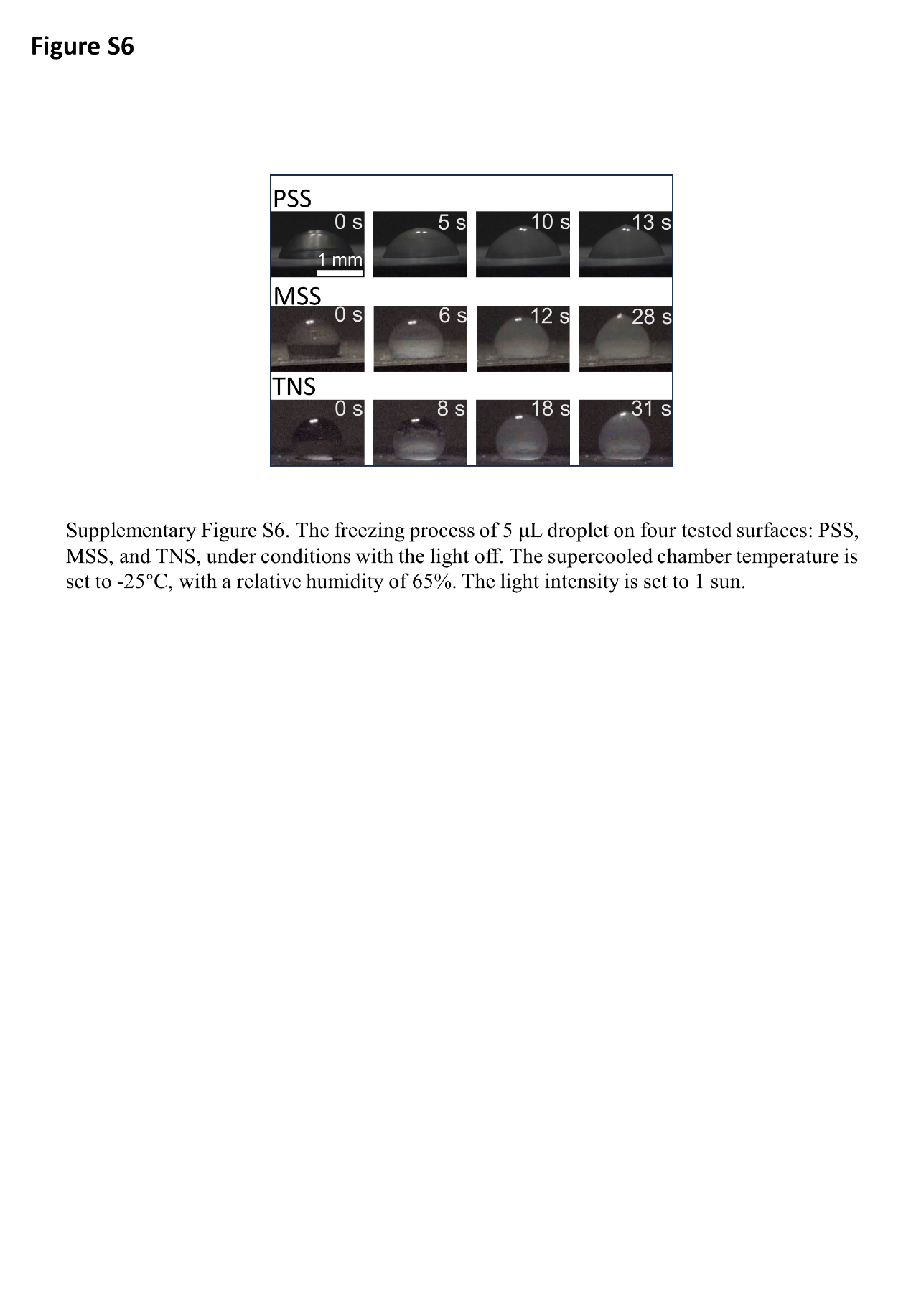


Supplementary Figure S6. The freezing process of 5 μL droplet on four tested surfaces: PSS, MSS, and TNS, under conditions with the light off. The supercooled chamber temperature is set to −25 °C, with a relative humidity of 65%. The light intensity is set to 1 sun.


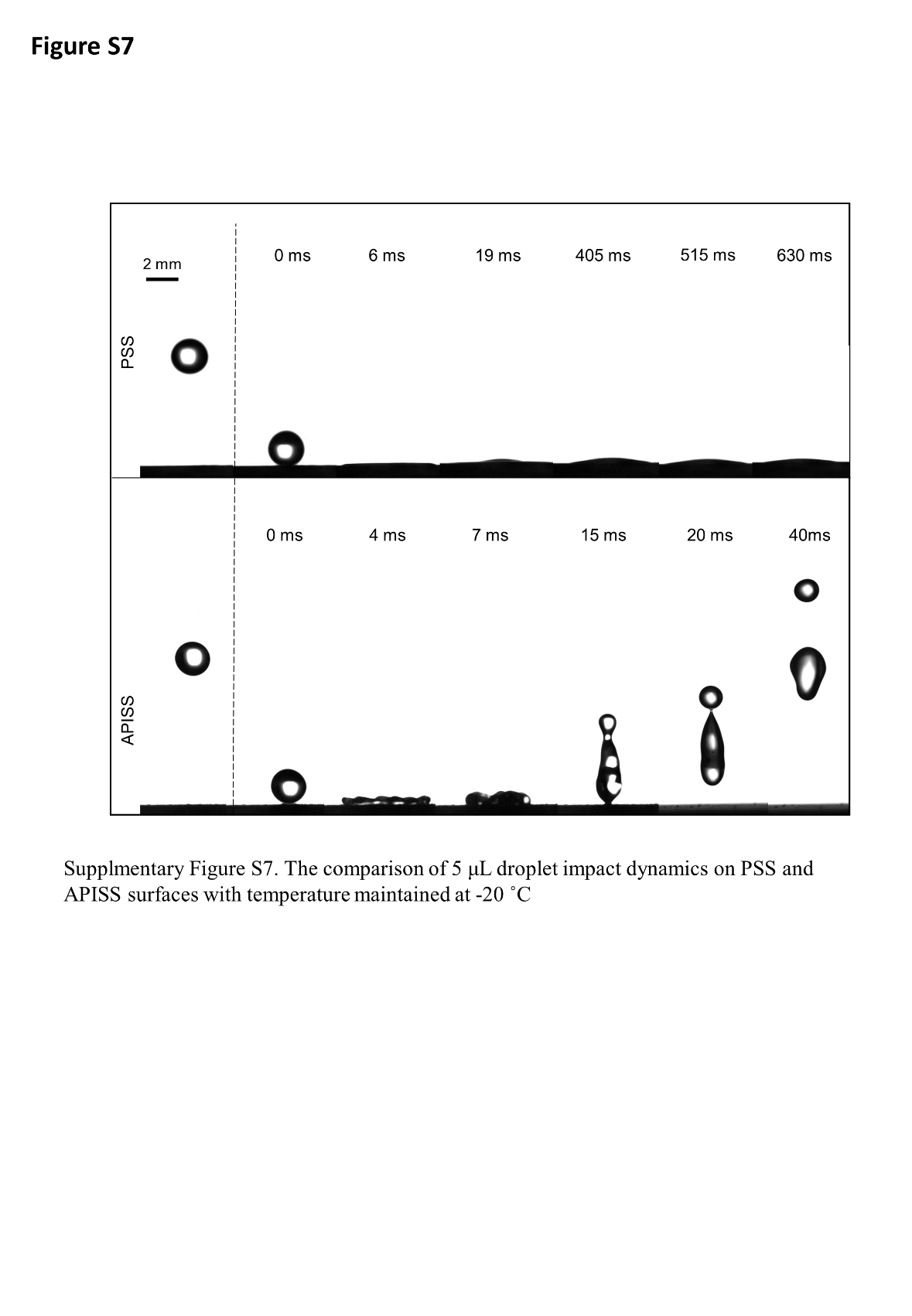


Supplementary Figure S7. The comparison of 5 μL droplet impact dynamics on PSS and APISS surfaces with temperature maintained at −20 °C.


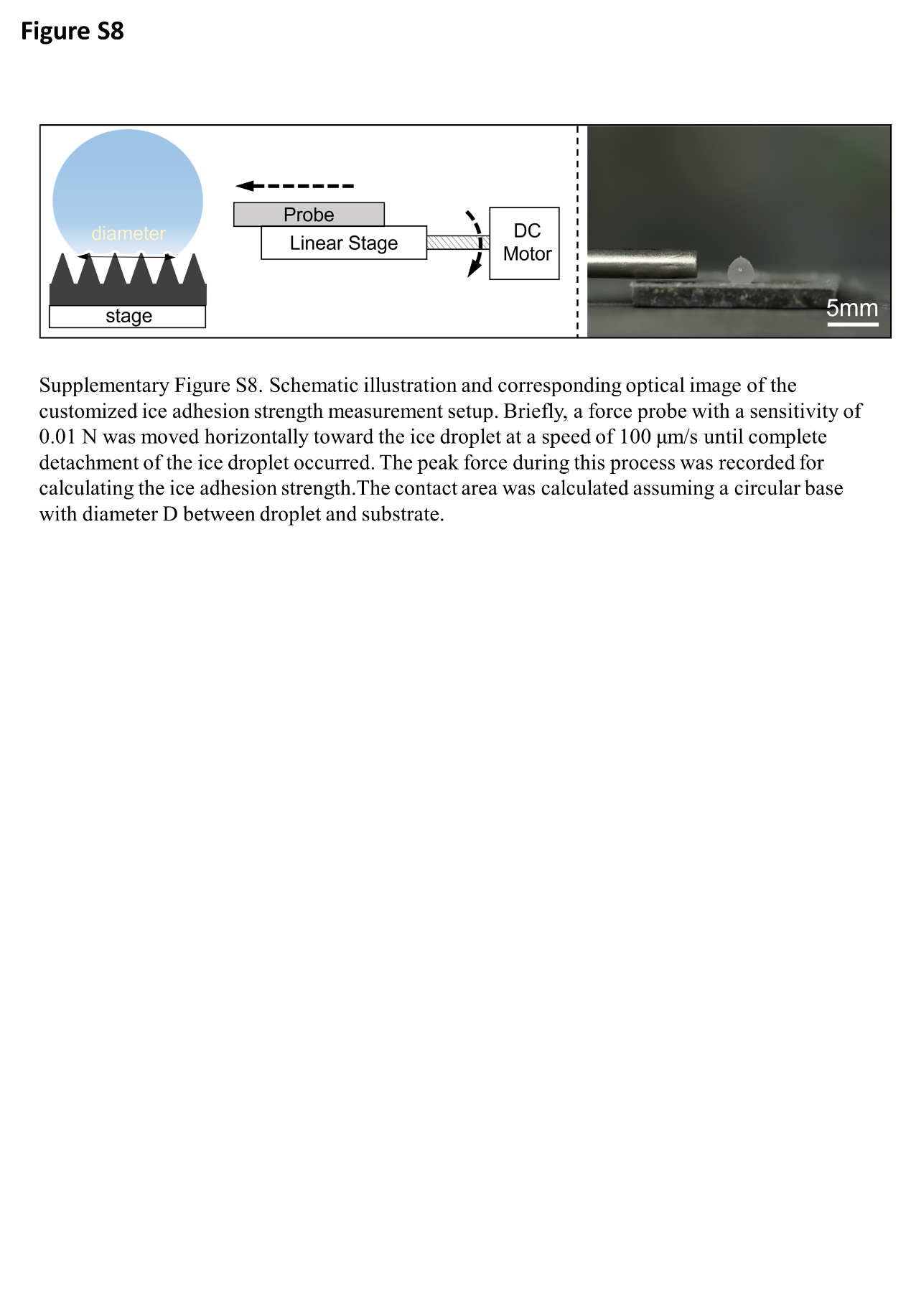


Supplementary Figure S8. Schematic illustration and corresponding optical image of the customized ice adhesion strength measurement setup. Briefly, a force probe with a sensitivity of 0.01 N was moved horizontally toward the ice droplet at a speed of 100 μm s^-1^ until complete detachment of the ice droplet occurred. The peak force during this process was recorded for calculating the ice adhesion strength. The contact area was calculated assuming a circular base with diameter D between droplet and substrate.


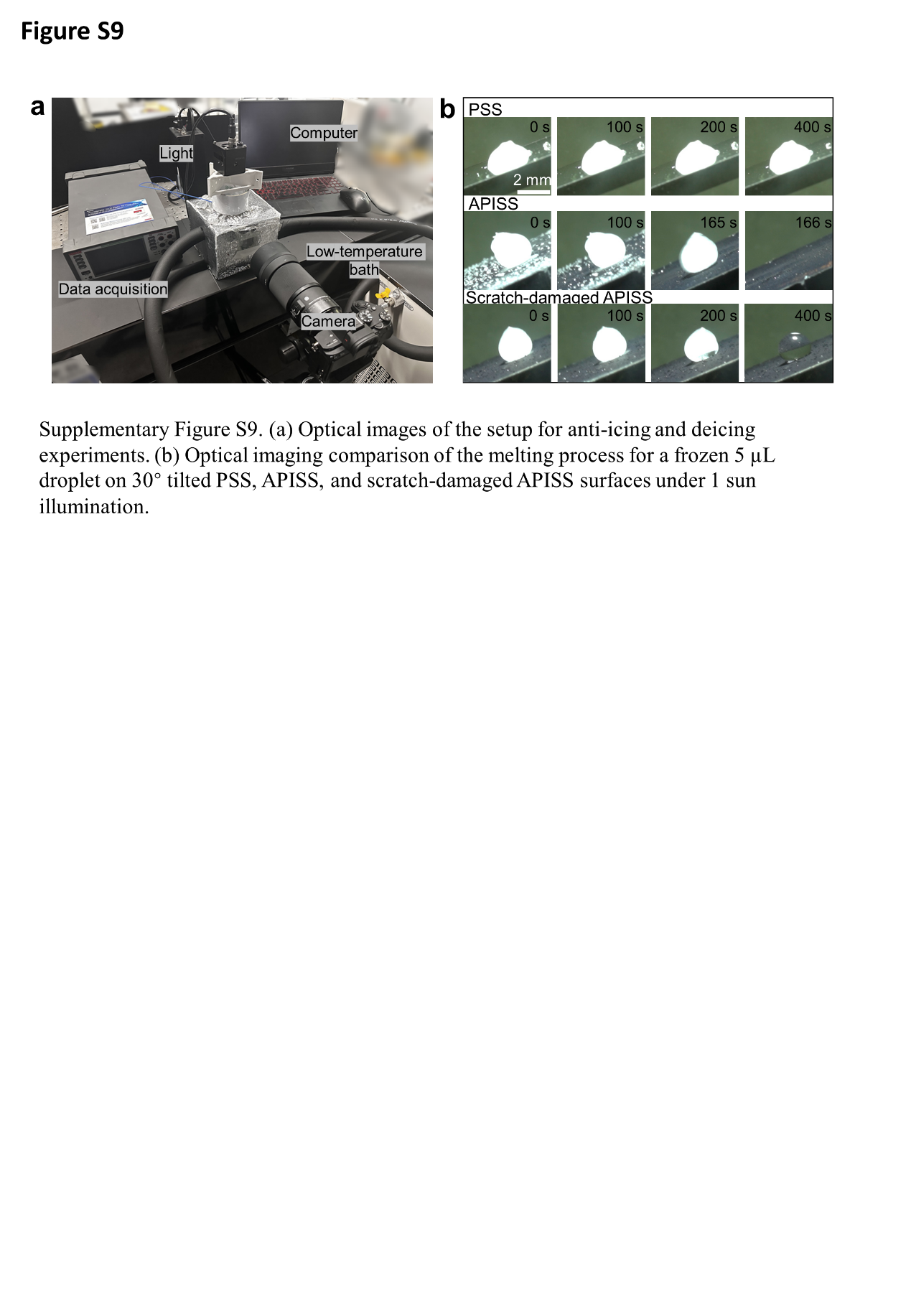


Supplementary Figure S9. (a) Optical image of the experimental setup. (b) Comparison of the melting processes captured by visible-light imaging for frozen droplets (5 µL) placed on PSS, APISS, and scratch-damaged APISS surfaces tilted at 30°, under 1 sun illumination.


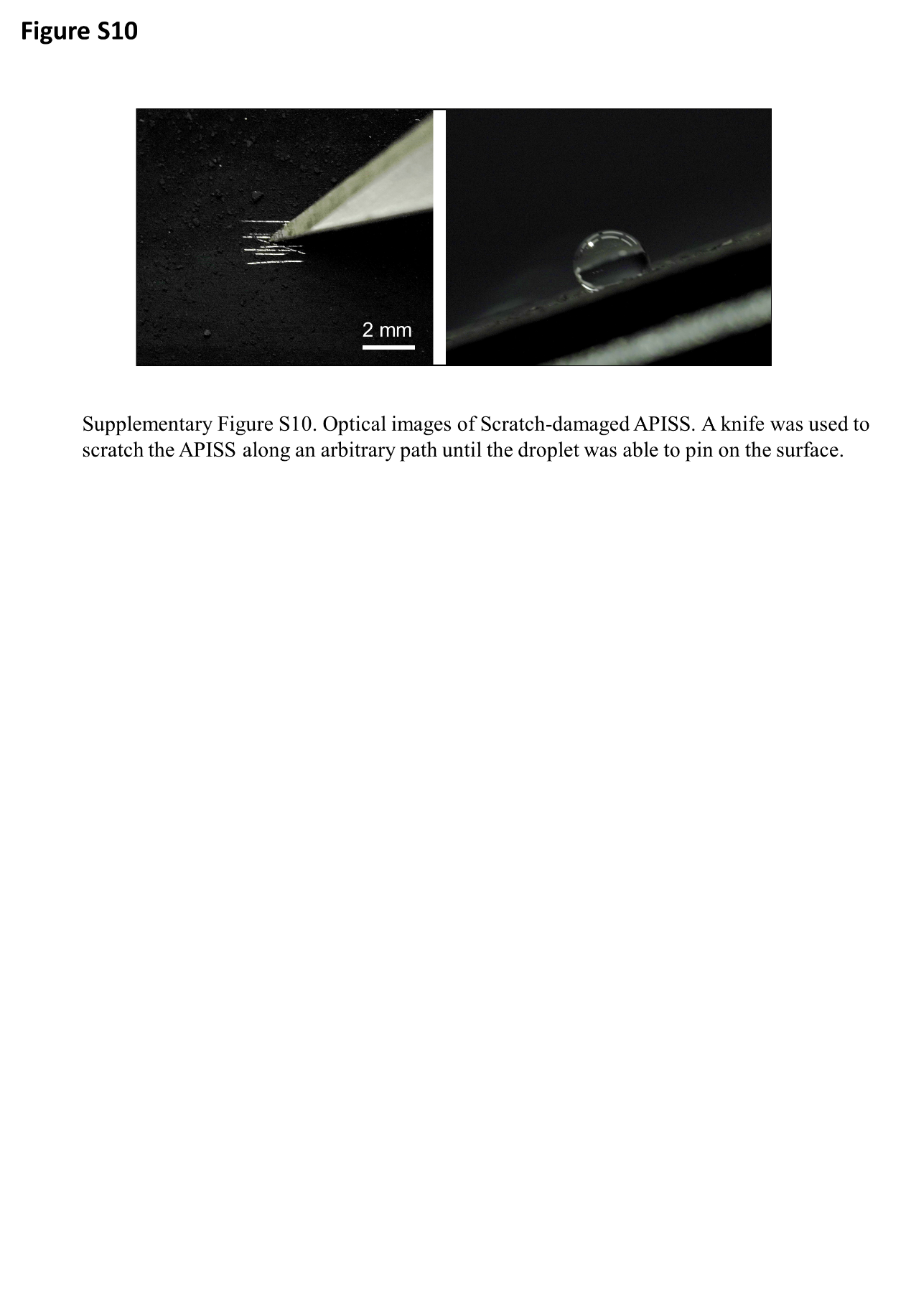


Supplementary Figure S10. Optical images showing the scratch-damage procedure of the APISS sample. A sharp knife was used to deliberately scratch the coating surface so that the water droplet could easily get pinned on the damaged region.


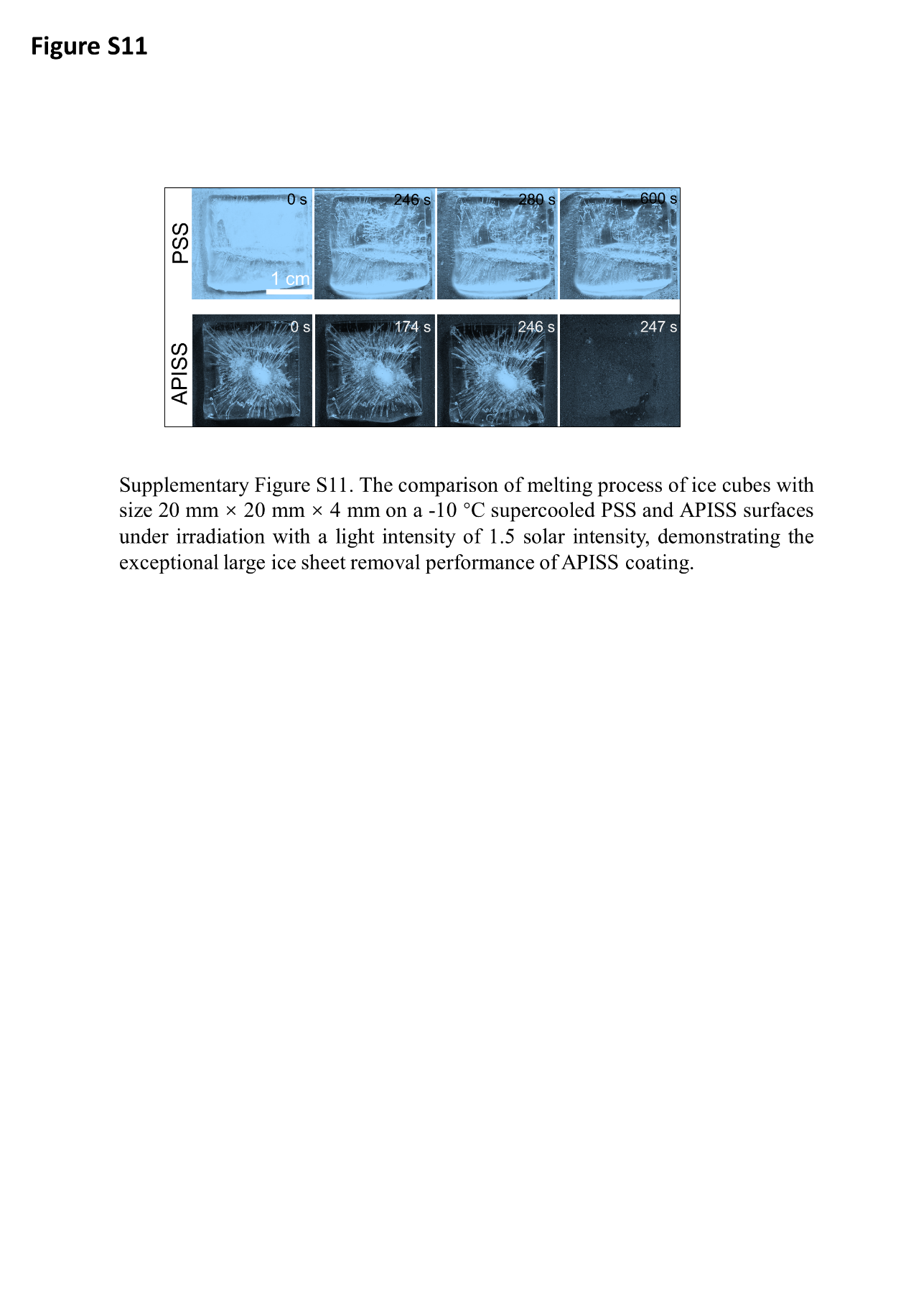


Supplementary Figure S11. The comparison of melting process of ice cubes with size 20 mm × 20 mm × 4 mm on a −10 °C supercooled PSS and APISS surfaces under irradiation with a light intensity of 1.5 solar intensity, demonstrating the exceptional large ice sheet removal performance of APISS coating.


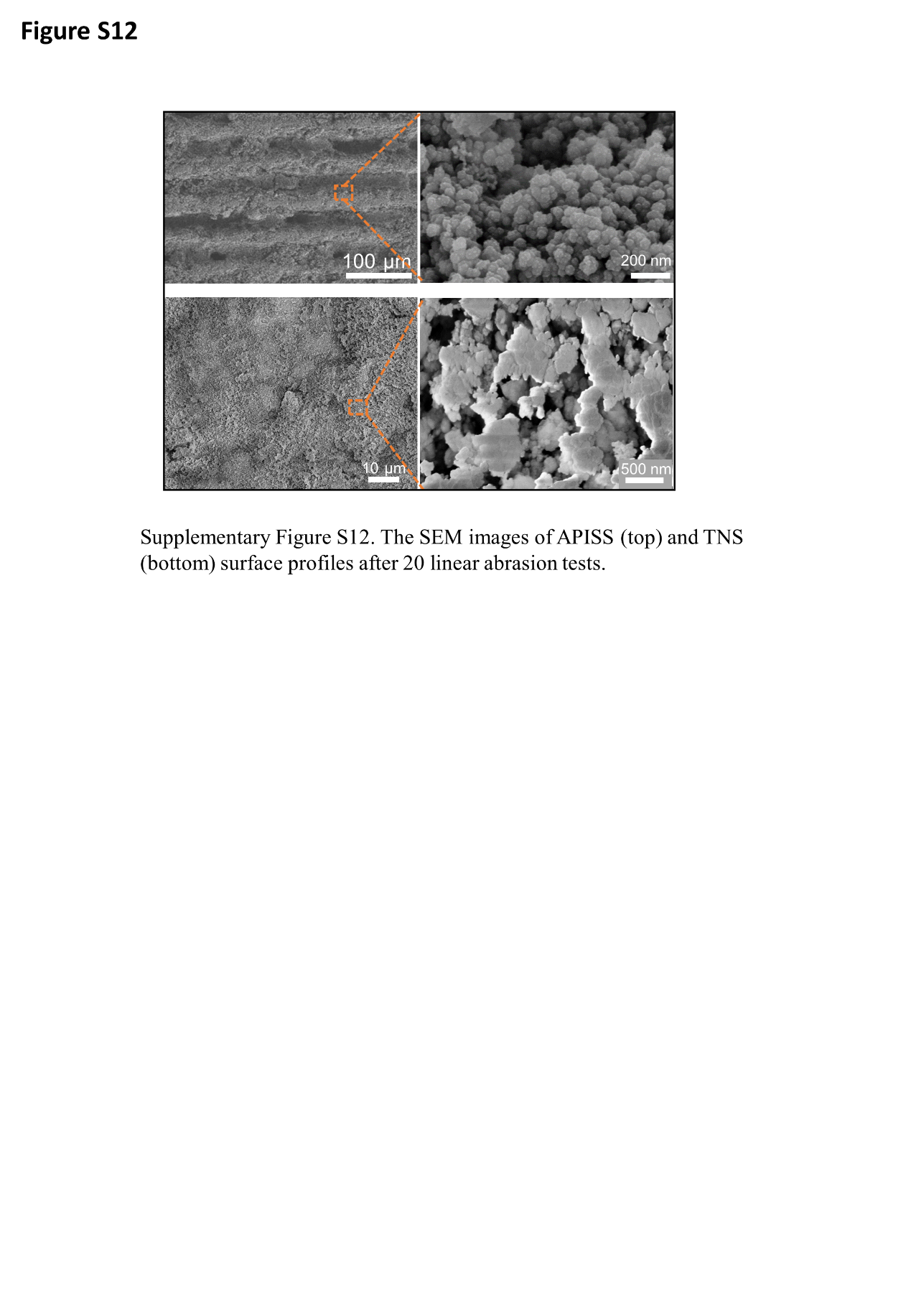


Supplementary Figure S12. The SEM images of APISS (top) and TNS (bottom) surface morphologies after 20 cycles of linear abrasion testing. The APISS shows well-retained nanoparticles embedded within the microscale structures, as the large-scale microgrooves effectively shield the fragile nanostructures from abrasion. In contrast, the TNS sample exhibits notable nanoparticle loss and surface smoothing after abrasion.


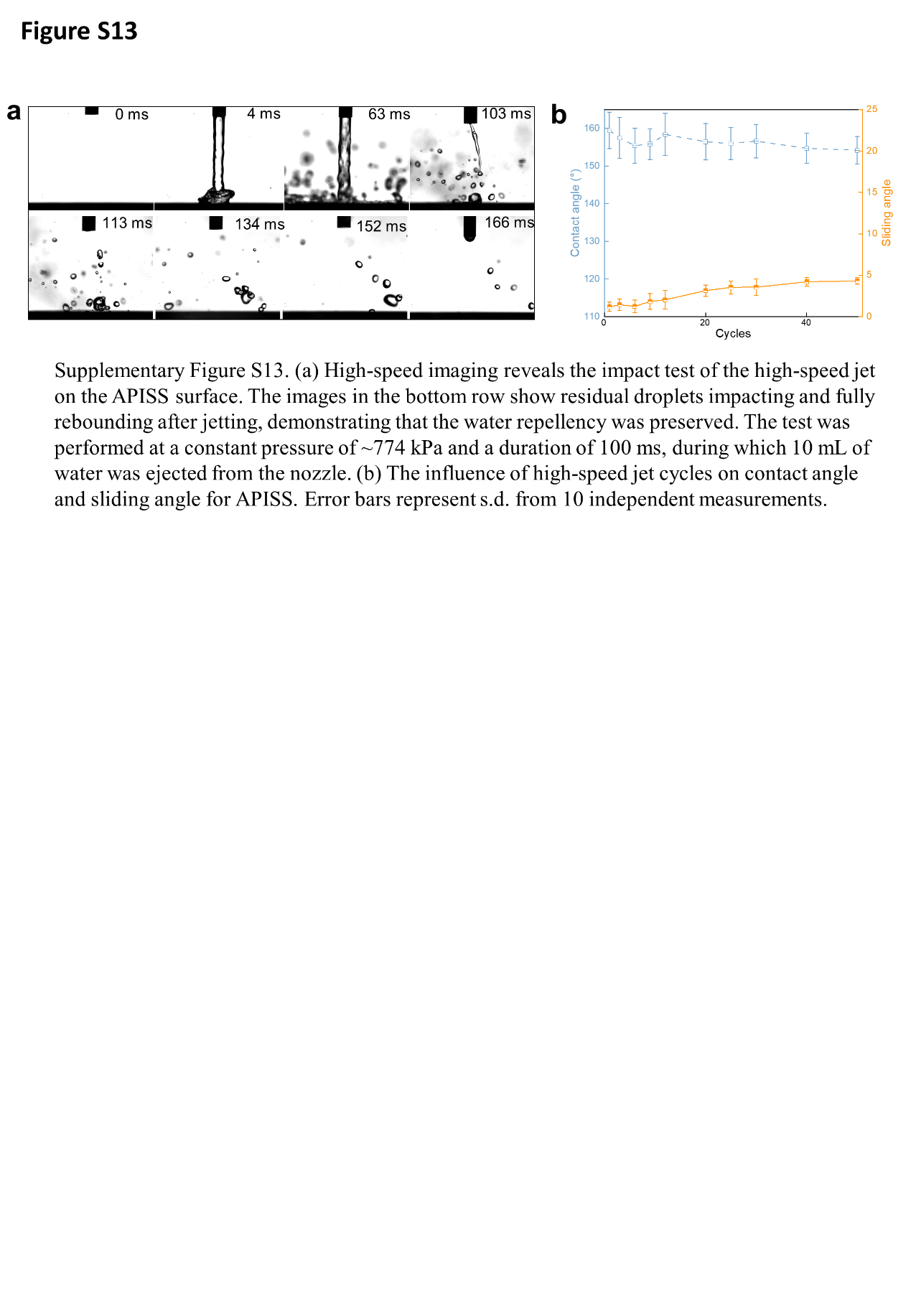


Supplementary Figure S13. (a) High-speed imaging reveals the impact test of the high-speed jet on the APISS surface. The images in the bottom row show residual droplets impacting and fully rebounding after jetting, demonstrating that the water repellency was preserved. The test was performed at a constant pressure of ~774 kPa and a duration of 100 ms, during which 10 mL of water was ejected from the nozzle. (b) The influence of high-speed jet cycles on contact angle and sliding angle for APISS. Error bars represent s.d. from 10 independent measurements.


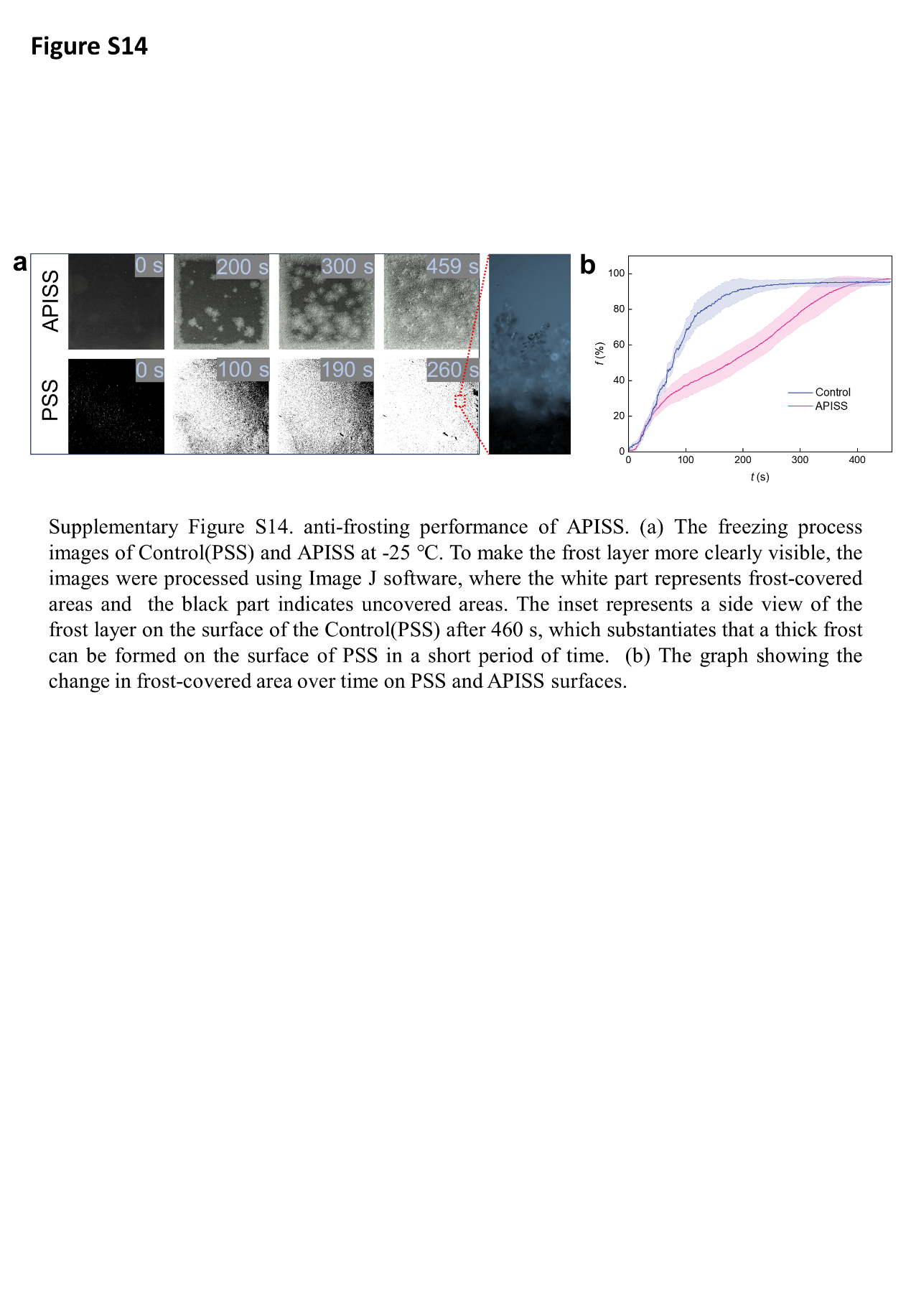


Supplementary Figure S14. The comparison of anti-frosting performance. (a) The images of freezing process for APISS and the control sample (PSS) at −25 ℃. To make the frost layer more clearly visible, the images were processed using Image J software, where the white part represents frost-covered areas and the black part indicates uncovered areas. The inset shows the side view image of the frost layer on the surface of the PSS sample after 260 s. (b) The graph showing the measured variation of the frost-covered area over time on PSS and APISS samples.


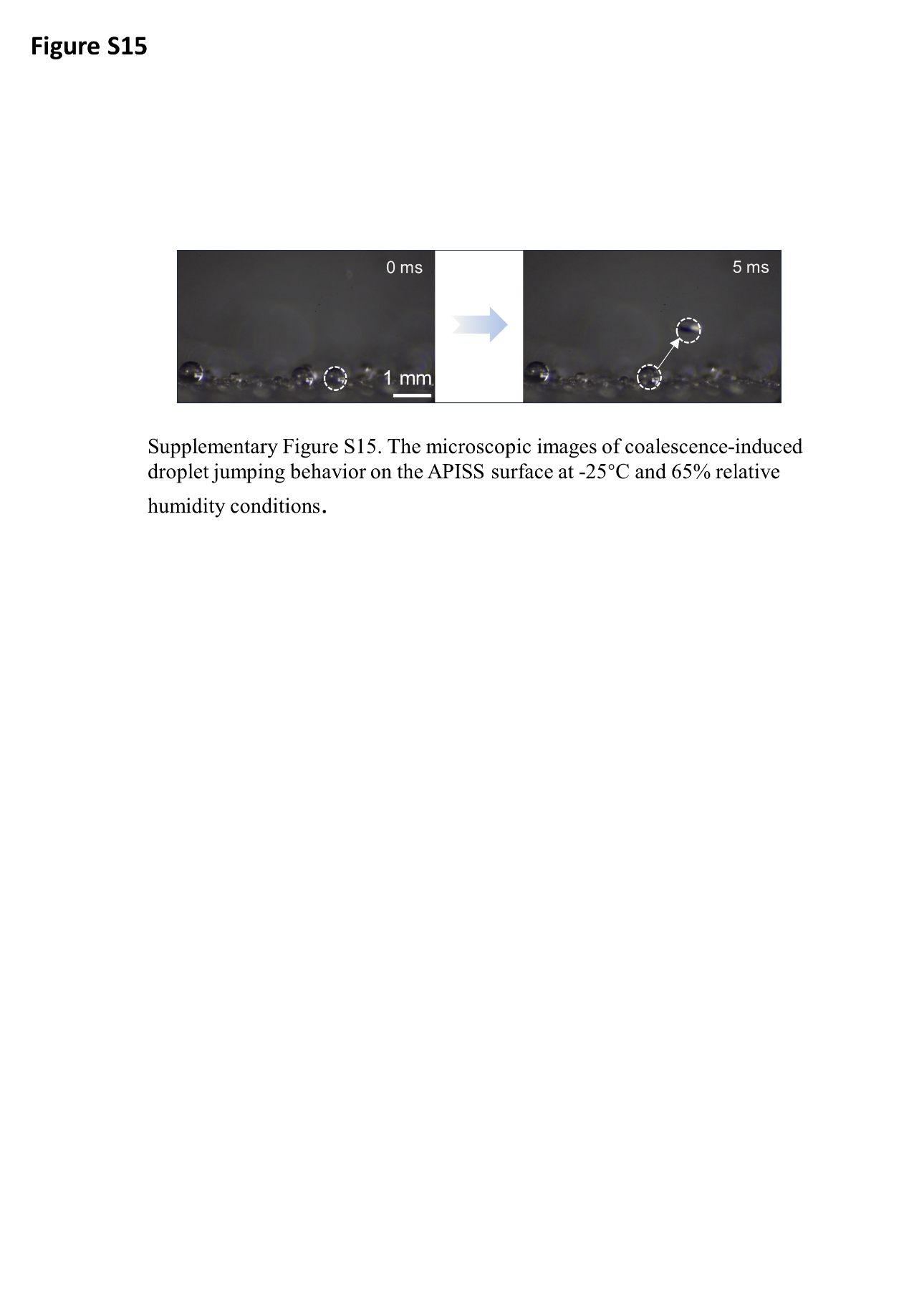


Supplementary Figure S15. The microscopic images of coalescence-induced droplet jumping behavior on the APISS surface at −25 °C and 65% relative humidity conditions.

**Supplementary Movies**

**Supplementary Movie S1**: The sliding angle dynamic test video.

**Supplementary Movie S2**: Infrared imaging comparison of the melting process for a frozen 5 µL droplet on 30° tilted PSS, APISS, and scratch-damaged APISS samples under 1 sun illumination.

**Supplementary Movie S3**: The shedding behavior of TiN coating without armored protective silica shell after one icing-deicing cycle.

**Supplementary Movie S4**: The icing-deicing cycle demonstrating exceptional mechanical stability of our APISS coating.

**Supplementary Movie S5**: Coalescence-induced droplet jumping phenomenon observed during the frosting process on APISS coating samples.

**References**

[1] P. Guo, Y. Zheng, M. Wen, C. Song, Y. Lin, L. Jiang, *Adv. Mater.* **2012**, *24* (19), 2642.

[2] Y. Hou, M. Yu, Y. Shang, P. Zhou, R. Song, X. Xu, X. Chen, Z. Wang, S. Yao, *Phys. Rev. Lett.* **2018**, *120* (7), 075902.
